# A Review of Development and Utilization for Edible Fungal Polysaccharides: Extraction, Chemical Characteristics, and Bioactivities

**DOI:** 10.3390/polym14204454

**Published:** 2022-10-21

**Authors:** Yujun Sun, Huaqi He, Qian Wang, Xiaoyan Yang, Shengjuan Jiang, Daobing Wang

**Affiliations:** 1 College of Life and Health Sciences, Anhui Science and Technology University, Fengyang 233100, China; 2 College of Agriculture, Anhui Science and Technology University, Fengyang 233100, China

**Keywords:** edible fungus, polysaccharides, extraction method, chemical composition, bioactivity

## Abstract

Edible fungi, commonly known as mushrooms, are precious medicinal and edible homologous gifts from nature to us. Because of their distinctive flavor and exceptional nutritional and medicinal value, they have been a frequent visitor to people’s dining tables and have become a hot star in the healthcare, pharmaceutical, and cosmetics industries. Edible fungal polysaccharides (EFPs) are an essential nutrient for edible fungi to exert bioactivity. They have attracted much attention because of their antioxidant, immunomodulatory, antitumor, hypoglycemic, and hypolipidemic bioactivities. As a result, EFPs have demonstrated outstanding potential over the past few decades in various disciplines, including molecular biology, immunology, biotechnology, and pharmaceutical chemistry. However, the complexity of EFPs and the significant impact of mushroom variety and extraction techniques on their bioactivities prevents a complete investigation of their biological features. Therefore, the authors of this paper thoroughly reviewed the comparison of different extraction methods of EFPs and their advantages and disadvantages. In addition, the molecular weight, monosaccharide composition, and glycosidic bond type and backbone structure of EFPs are described in detail. Moreover, the in vitro and in vivo bioactivities of EFPs extracted by different methods and their potential regulatory mechanisms are summarized. These provide a valuable reference for improving the extraction process of EFPs and their production and development in the pharmaceutical field.

## 1. Introduction

Edible fungi, commonly experienced as mushrooms, are one of the most popular beneficial or healthy foods in the daily diet due to their wide variety and favorable culinary and nutritional value. In addition, they also have valuable medicinal functions [1]. Biomass or specific extracts from mushrooms’ developmental stages, including fruiting bodies, sclerotia, mycelium, and spores, have been used as beneficial foods or dietary supplements [2,3]. Mushrooms are an abundant source of many bioactive products, such as polysaccharides, fibers, ergosterols, flavonoids, lectins, terpenoids, and proteins [4]. They are rich in macro and micronutrients as well as minerals, vitamins, and amino acids [5]. These resulting bioactive secondary metabolites or nutrients are increasingly being extracted and encapsulated or tableted as a functional dietary supplement or regulator. The regular intake of these supplements can enhance the body’s immune function, resulting in enhanced resistance to disease and faster recovery [4].

EFPs are one of the most common functional active compounds in mushroom mycelium, fruiting bodies, and fermentation broth [6]. EFP is a type of natural macromolecular polymer with a complicated structure and a high molecular weight created by the glycosidic bonding of at least ten monosaccharide molecules. There are numerous EFPs, including heteropolysaccharides rich in monosaccharides such as fucose, galactose, mannose, and xylose, glycogen-like glucans as storage components, and structural cell wall polysaccharides [7]. EFP has been linked to various biofunctions, including blood glucose and lipid regulation, scavenging free radicals, antitumor, anti-fatigue, anti-aging, immunomodulation, and gastric mucosa protection [8]. Furthermore, since EFPs have less glycoprotein content and fewer toxic side effects, they are increasingly employed in food, pharmaceuticals, and cosmetics [9].

EFP bioactivity is affected by the fungal species, culture conditions of production, and extraction method. Various traditional and potential extraction methods have been devised and proposed as extraction technology has advanced [10]. Generally, crude extracellular polysaccharides can be directly extracted from fungal fermentation media by the ethanol-precipitation method [11]. The extraction of intracellular fungal polysaccharides is more based on the fruiting body or mycelium of fungi, and the extraction methods are also rather variable [12]. Before the extraction of intracellular EFPs, the raw materials were subjected to a series of pretreatments, including washing, drying, crushing, and defatting. Therefore, extraction methods significantly influence the yields and characteristics of polysaccharide products and their bioactivities [13]. Each method has its advantages and disadvantages. Hot water extraction (HWE), for example, is one of the most often utilized extraction procedures due to its convenient operation, basic equipment, and simple implementation. However, the method is time-consuming and inefficient [14,15]. Dilute alkali-assisted extraction, another popular method, can improve extraction efficiency and shorten the separation time of acid EFPs, but it is easy to disrupt the structure of EFPs [16].

Therefore, the authors of this review systematically summarized and compared the current extraction method, chemical composition, in vitro and in vivo bioactivities, and regulatory mechanism of EFPs. These descriptions can serve as a development idea or a reference for the comprehensive utilization of EFPs.

## 2. Extraction Method of EFPs

**Figure 1:**
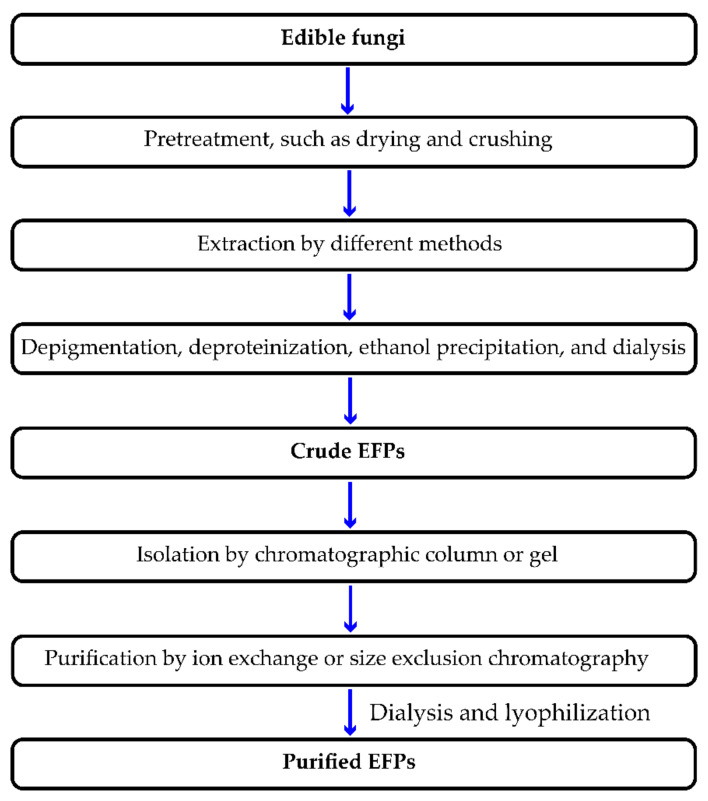
Preparation process of EFPs.

To efficiently extract polysaccharides, mushroom fruiting bodies or cultured mycelium are typically isolated and purified through several procedures. The extraction phase is the most important, since it controls the recovery rate, monosaccharide composition, molecular weight, structure and spatial configuration, and, most importantly, the bioactivity of the isolated polysaccharide [17]. Since most functional polysaccharides in mushrooms are water-soluble, they can be extracted using water extraction assisted by physical techniques (e.g., heating, ultrasound, and microwave) [18]. Although most polysaccharides are polar molecules and are highly soluble in water or alkaline solutions, several insoluble polysaccharides, including some β-glucans, require longer extraction times, higher temperatures, or pressures for effective extraction [7]. Depending on various factors, including the environment in which edible fungi grow and the characteristics of the strain, it may be necessary to perform a series of pretreatment steps to separate EFPs, such as preliminary centrifugation to separate the insoluble components in the raw materials. In addition, using reagents such as trichloroacetic acid or protease can precipitate proteins. As representative of conventional extraction methods, HWE (Table 1) is the most commonly used in separating and extracting EFPs due to their simple operation and low cost. Presently, many advanced extraction techniques help in the extraction and recovery of EFPs from mushrooms, such as ultrasonic-assisted extraction (UAE), microwave-assisted extraction (MAE), enzyme-assisted extraction (EAE), ultrasonic-microwave synergistic extraction (UMSE), subcritical water extraction (SWE), pulsed electric field-assisted extraction (PEFAE), and comprehensive extraction techniques (Table 1) [5]. After precipitation with cold ethanol, the precipitated EFPs are dialyzed with water to remove the unwanted low molecular weight compounds (Figure 1). In addition, more purification stages can be carried out, such as ion exchange chromatography or size exclusion chromatography (Figure 1).

### 2.1. HWE

HWE is ideal for laboratory or large-scale industrial synthesis of EFPs because it is easy to use and inexpensive [19]. Many researchers have used HWE to prepare EFPs. Hou et al. [20] optimized the extraction process of crude polysaccharides from wild edible BaChu mushrooms based on response surface methodology. The optimal extraction conditions were as follows: 94 °C of extraction temperature, 10 h of extraction time, and 1:6 solid–liquid ratio. Under optimized conditions, the yield of polysaccharides was 8.73%. After removing the protein, crude polysaccharide was obtained. Zhang et al. [21] crushed cultured *Cordyceps Sinensis* and performed two extractions (repeated twice) in boiling water for 2 h. Yin et al. [22] extracted *Flammulina velutipes* mycelium powder in a hot water bath (repeated twice for 4 h each time, 95 °C). After precipitation, deproteinization, and decolorization, the crude *Flammulina velutipes* mycelium polysaccharides (CFVPs) were obtained, and the yield was 2.2%. Morales et al. [23] discovered that by extracting the mushroom powder with hot water at 98 °C for 1 h, a higher concentration of β-D-glucan extract could be achieved after tweaking the extraction settings. Dey et al. [24] added *Pleurotus florida* blue variant to boiling water for 6 h extraction to obtain crude polysaccharide. Han et al. [25] pretreated fruit bodies from *Sarcodon aspratus* with 95% ethanol for 1.5 h, which was followed by an extraction in boiling water for 1.5 h, and repeated 3 times, to prepare crude polysaccharides. Wang et al. [26] pretreated the fruiting bodies of *Lentinus edodes* with 95% ethanol for 24 h and then extracted them in boiling water twice, 1 h each time. After a series of precipitation and impurity removal steps, crude polysaccharide (SLNT) in 0.9% yield was obtained. Kanwal et al. [27] mixed the fruit body powder of *Dictyophora indusiata* with boiling water at a ratio of 1:30 (*w*/*v*) and then extracted it for 2 h to obtain crude polysaccharide (DIP). Mfopa et al. [28] extracted the dried and crushed fruiting body powders of *Ganoderma applanatum* in boiling water for 3 h and treated them with 95% ethanol at 4 °C for 24 h to obtain polysaccharides. Wang et al. [29] treated the dried *Poria cocos* Wolf powder with petroleum ether for 24 h at room temperature, extracted them with a ratio of 20:1 (mL/g) in boiling water for 2 h, and then precipitated with four volumes of ethanol at 4 °C to obtain *Poria cocos* Wolf polysaccharide (PCP-H). The yield of the polysaccharide obtained by this method was about 3.5%.

HWE is a simple and feasible extraction technology, but it has several evident drawbacks, including a long processing time, high working temperature, high energy demand, low extraction efficiency, and it is highly unfriendly in the extraction process of some rare edible fungi [19,30]. Additionally, organic chemicals such as ethanol or acetone employed in the pretreatment procedure in HWE may have a long-lasting negative impact on the bioactivity rate of EFPs [31]. Longer processing durations may result in the release of large amounts of undesired cell wall components (such as pectin), tissue or cell debris and higher purification costs [19,30]. Several studies have demonstrated that yield or purity declines with increasing temperature over the optimum level [32]. For example, Su et al. studied the effect of HWE extraction temperature on the physicochemical properties of EFPs from *Grifola frondose* [32]. They found that 121 °C was the optimal extraction temperature (the highest yield was 3.35%), and higher temperatures caused polysaccharide molecular weight to decrease. Additionally, high temperatures may lead to the degradation of β-glucan and the unwinding of the triple helix structure [33]. Many auxiliary or novel extraction techniques have been proposed to compensate for the shortcomings of HWE extraction.

### 2.2. UAE

**Figure 2:**
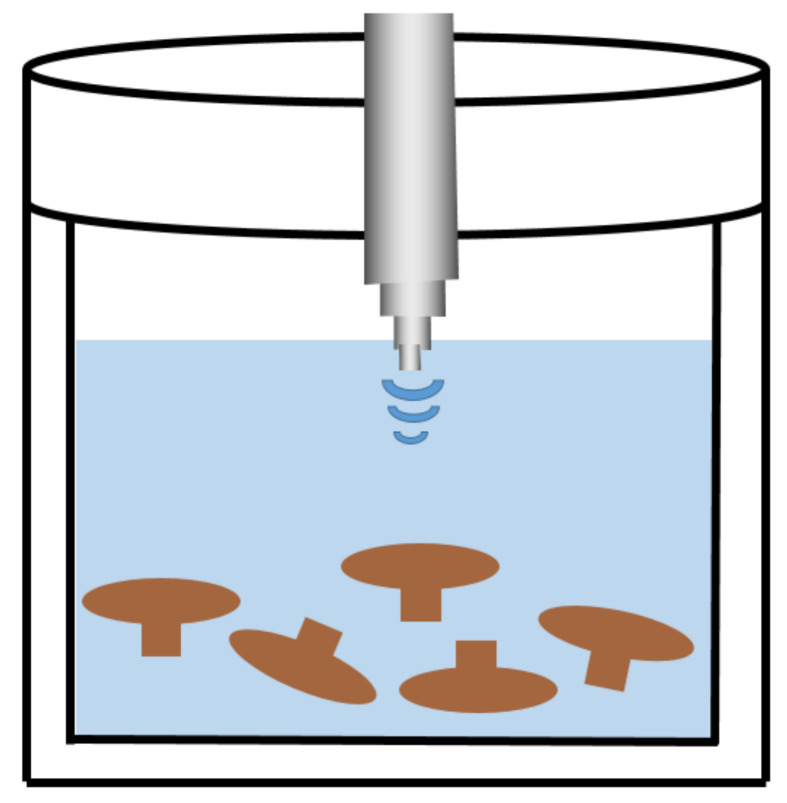
EFP extraction by UAE.

After HWE, UAE is another extraction method that is widely used to extract EFP, and its extraction diagram is exhibited in Figure 2. UAE releases a large amount of energy to generate microjets, shock waves, and high shear forces by utilizing the cavitation effect produced by the formation of acoustic cavitation in the solvent and the collapse of asymmetric microbubbles. As a result of improved osmotic and capillary effects, reduced particle size, and potential cell wall disintegration by hydrodynamics, extraction yield and efficiency are increased [34,35]. The thermal effect of UAE is caused by the release of high local energy during bubble collapse, promoting the diffusion of polysaccharides into the solvent. The microstructural analysis of polysaccharide residues extracted by UAE revealed that explosive destruction caused by acoustic cavitation and changes in the cellular matrix could lead to considerable changes in the residue structure, which favored the diffusion of large-MW polysaccharides [36]. Due to the rise in bubble size in the UAE, using water at lower vapor pressures (30–100 mmHg) and temperatures (30–50 °C) can increase cavitation intensity [36]. Low frequency (20–100 kHz) UAE offers the benefits of decreased solvent and energy usage, high processing throughput, and rapid processing times (usually under 1 h) [37]. Additionally, increasing ultrasonic power can decrease extraction time and temperature to prevent polysaccharide thermal deterioration [38]. Alzorqi compared the extraction efficiency of three different extraction methods for β-D-glucan from artificially cultivated mushrooms and discovered that UAE had a higher extraction rate, less energy consumption, and higher polysaccharide purity compared to Soxhlet extraction (SE) and HWE [36]. Furthermore, the monosaccharide compositions of polysaccharides by UAE and SE were similar, with high proportions of glucose and galactose, followed by mannose and fucose, whereas HWE had a higher glucose content but a smaller proportion of galactose and fucose. UAE-extracted polysaccharides also showed superior DPPH radical scavenging and iron-reducing capacity when compared to HWE and SE extraction. Cheung et al. [39] used the UAE method (20 kHz frequency and 130 W) and extracted with a solid-to-liquid ratio of 1:3 (*w*/*v*) at 45–50 °C for 30 min to prepare medicinal fungi polysaccharides. Zhao et al. [40] used UAE to extract polysaccharides from the base of *Flammulina velutipes*, and they obtained optimal extraction parameters by the response surface method (RSM): 680 W of ultrasonic power, 19.8 min of extraction time, and 28:1 mL/g the liquid–solid ratio. The yield of polysaccharide obtained by this method was 16.20%. *Poria* polysaccharide (PCP-U) was obtained by extraction in a 200 W ultrasonic bath for 1 h at a liquid–solid ratio of 20:1 (mL/g) by Wang et al. [29]. Chen et al. dissolved *Grifola frondosa* powder in hot water (1:30 g/mL) and ultrasonically extracted at 50 kHz for 30 min, then further extracted in a water bath (90 °C) for 3 h, and finally precipitated with absolute ethanol to obtain polysaccharides [41]. Yan et al. [42] found that different ultrasonic treatment intensities (200, 400, and 600 W) lowered the intrinsic viscosity and MW of polysaccharides and shortened their MW distribution, but it had no impact on their monosaccharide composition or preliminary structure. More importantly, the UAE treatment effectively increases the bioactivity of polysaccharides while also being gentler, more ecologically friendly, and more effective.

UAE can reduce extraction time and solvent usage, which are considered major limitations of any other extraction process. A lower extraction temperature during extraction reduces energy consumption, improves yield, and is more favorable for preserving the bioactivity of polysaccharides [43,44]. Accordingly, UAE has been proven to be an environmentally friendly technology. However, measuring temperature increases in the UAE can be challenging, which could result in inconsistent results. Additionally, it has been reported that ultrasonic treatment easily leads to a drop in polysaccharide molecular weight (MW) and degradation of chemical structure [45]. The MW of *Hohenbuehelia* polysaccharide, for instance, was only 1.14 kDa when extracted by UAE compared to 3.87 kDa when extracted by HWE [45]. Similarly, the low-MW proportion of *Flammulina velutipes* polysaccharides by UAE was increased, and the triple helix structure was destroyed.

### 2.3. MAE

**Figure 3:**
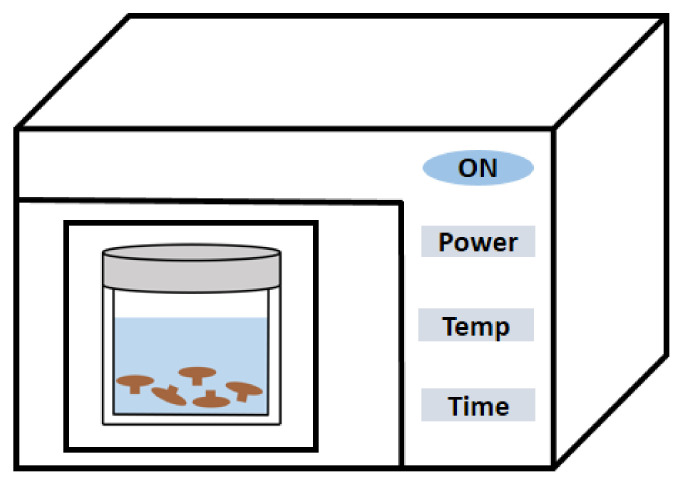
EFP extraction by MAE.

Over the past two decades, MAE has been crucial in the last two decades for the extraction and drying of natural products [46]. Microwaves are a non-contact heat source that produces thermal energy by breaking down cell walls and releasing polysaccharide molecules by ionic conduction between a solvent and solute. Changes in polar molecular orientation due to high-frequency alternating electric fields induce molecular vibrations and rotations, resulting in enhanced collision frequency, increased internal pressure, and abrupt temperature rise, ultimately causing cell rupture and solvent diffusion through the cell barrier [47]. Flexibility, minimal solvent use, rapid processing, and high extraction yield are all benefits of MAE [48]. Microwave power, processing temperature, and extraction time are all crucial parameters to consider when extracting EFPs from mushrooms using MAE; its extraction diagram is exhibited in Figure 3. Wang et al. [29] placed the dried and defatted *Poria cocos* Wolf powder in a microwave extractor for 2 min to obtain *Poria cocos* Wolf polysaccharides (PCP-M) (20:1 mL/g, 2450 MHz, 800 W). Gil-Ramírez et al. [49] optimized MAE operating conditions using shiitake mushroom as a model strain to extract EFPs. The optimal conditions (180 °C and 30 min) were applied to the extraction of EFPs from other mushroom species, such as *Agrocybe aegerita*, *Cantharellus cibarius*, *Hypsizygus marmoreus*, and *Morchella conica*. The average yield of polysaccharides was between 12.1 and 17.8%, and the polysaccharide content was 51.1–68.2%. Wang et al. [29] placed the dried and defatted *Poria cocos* Wolf powder in a microwave extractor (2450 MHz, 800 W, 2 min) and then extracted *Poria cocos* polysaccharide with a liquid–solid ratio of 20:1 (mL/g). Zhu et al. [50] prepared *Cordyceps gunnii* mycelia polysaccharide by the MAE method (280 W, 70 °C, 5 min) after combining dried *Cordyceps gunnii* mycelia powder with deionized water (1:20, *w*/*v*). Compared with other extraction methods from the same period (cold water extraction, HWE, UAE, and enzyme-assisted extraction), MAE produces higher yields with the shortest processing time.

The inhomogeneity of the heating is one of the critical drawbacks of MAE in comparison to HWE and UAE [51]. Extremely high microwave power interferes with molecular interactions, lowering the polysaccharide extraction rate [47]. Longer extraction times also promote the absorption of microwave energy and subsequent heat accumulation in the medium, which causes polysaccharides in the extraction media to degrade. To improve the drawbacks in the MAE process, Xu et al. used ultrasonic-microwave synergistic extraction (UMSE) to achieve higher extraction rates and shorter processing times [47,52]. However, polysaccharide extracts with high turbidity may be produced during UMSE, which is possibly due to the release of cellular debris during protein coagulation and thermal accumulation [47]. Thankfully, using UMSE may improve the bioactivity of polysaccharides in some cases. For instance, the purified *Tricholoma mongolicum* Imai polysaccharide produced by UMSE demonstrated more remarkable performance in terms of DPPH and OH- radical scavenging activities and higher antioxidant activity with increasing MW [51].

### 2.4. EAE

Enzymes can efficiently catalyze the hydrolysis and degradation of the fungal cell wall matrix to release bioactive compounds enclosed within the cell. EAE usually does not destroy the three-dimensional molecular structure of polysaccharides, thus helping to maintain its bioactivity [53]. In addition, EAE has the advantages of convenient operation, high specificity, environmental protection, high efficiency, low energy requirement, and mild reaction [8]. The extraction efficiency of EAE also depends on the type and concentration of enzyme, temperature, pH, reaction time, and liquid–solid ratio. Among them, the temperature is an essential factor for EAE, and an appropriate temperature increase will improve the energy of the reactants, the reaction rate, the number of cavitation bubbles, the contact surface area, the diffusivity of the solvent into the cell, and the solubility and desorption of the polysaccharide from the cell. However, extreme temperatures may lead to enzymatic inhibition and the thermal decomposition of polysaccharides [54]. Additionally, environmental factors also easily affect enzyme activity (e.g., temperature, dissolved oxygen, nutrient availability). Cellulase is a commonly used enzyme in the extraction process of EFPs [54]. Wang et al. [29] used a cellulase concentration of 1.0% at a liquid–solid ratio of 20:1 (mL/g) to extract *Poria cocos* Wolf polysaccharide (1 h). Zhu et al. [50] mixed dried *Cordyceps gunnii* mycelia powder with 2% solution containing 0.5% cellulase to extract EFPs. Zhao et al. [8] reported the application of cellulase in the extraction of *Tricholoma mongolicum* Imai polysaccharide, and the obtained polysaccharide purity (80.1%) was higher than that of UAE-MAE synergistic extraction (73.3%), but the latter exhibited a higher yield (23.7%). The cellulase-extracted polysaccharide was purified with a maximum MW of 269 kDa, and the highest DPPH and OH- radical scavenging activities of 78.5% and 84.4% at 2 mg/mL were exhibited, respectively. Multiple enzymes (such as cellulase, papain, pectinase, and protease or trypsin) are used in combination in many reports to help release polysaccharides and improve the extraction rate [53]. Yu et al. used complex EAE (cellulase:papain = 2:1, 1.5% of concentration, 30 mL/g of liquid-to-solid ratio, 50 °C, 138 min) accompanied by UAE (360 W, 20 min) to extract EFPs from *Armillaria mellea* mushroom, and finally, they obtained a yield of 40.56% [55]. The high-MW purified Lentinus edodes polysaccharide (644.7 kDa) obtained by complex EAE showed stronger anti-HCT-116 and HeLa cell proliferation effects than low-MW polysaccharides [56]. The study by Fan et al. [57] showed that *Grifola fondosa* EFPs extracted by complex EAE method had lower MW (233.5 kDa) but exhibited stronger antioxidant activity compared with single EAE and HWE. However, the higher cost is one of the main drawbacks of EAE [53].

### 2.5. Alkali/Acid-Assisted Extraction (AAE)

Generally, NaOH/KOH and HCl/(NH_4_)_2_C_2_O_4_ were used in AAE to promote the release of EFPs during extraction [58]. They are usually performed in sequential extraction steps following HWE to maximize the recovery of EFPs. Acid or alkaline treatment results in disruption of the cell wall and the cleavage of crude fibrous structural hydrolytic bonds in the cell wall between proteins and glucans (such as bonds to O-linked side chains), allowing the release of intracellular polysaccharides, extraction of acid or base solubility ingredients, and transformation of water-insoluble components into water-soluble components [59,60]. These contributed to the higher yield of AAE compared to HWE. NaBH_4_ is typically added during alkaline-assisted extraction to prevent oxidation [61]. Wang et al. [26] added 2% NaOH and 0.05% NaBH to the *Lentinus edodes* fruit body residue after boiling water extraction and treatment for 24 h. Then, the mixture was neutralized by 5% CH_3_COOH (Hac) and precipitated with 95% ethanol to obtain lentinine-extracted crude polysaccharide (JLNT) with a yield of 4.8%. Yang et al. [62] degreased mushroom fruiting bodies with 95% ethanol (1:10, *w*/*v*), extracted it with boiling water (1:20, *w*/*v*) three times (4 h each time), and then extracted with 0.5 M NaOH and a trace amount of NaBH_4_ at 80 °C for 3 h × 2 and 2 h × 1 (1:20, *w*/*v*) to obtain alkali-soluble polysaccharide. Nandi et al. [63] placed the washed fruit of *Russula albonigra* (Krombh.) Fr. in 4% NaOH for 1 h (100 °C) to obtain crude polysaccharide samples. Wang et al. [60] extracted the pretreated *Pellinus linteus* mycelium powder at 95 °C for 8 h (repeated twice). They then used 1% (NH_4_)_2_C_2_O_4_ (*w*/*v*) extracted twice under the same conditions and finally extracted with 1.25 M NaOH/0.05% NaBH_4_ for 3 h at room temperature (repeated twice) to obtain the polysaccharide samples. Decha et al. successively used hot water, 1% (NH_4_)_2_C_2_O_4,_ and 1.25 M NaOH/NaBH_4_ solution to extract polysaccharides from *Pleurotus sajor-caju* [64]. Compared with alkaline-extracted polysaccharides, polysaccharides extracted with ammonium oxalate had the highest yield and carbohydrate content while containing fewer protein impurities. Szwengiel et al. [65] used acid-assisted extraction to extract polysaccharides from *Pleurotus ostreatus*. During the extraction process, the linkages between proteins and polysaccharides were broken down by acid. Compared with HWE, its β-D-glucan yield (3.5%) was significantly increased seven-fold. Although AAE can increase the yield of EFPs, alkali/acid will cause a break of hydrogen bonding, O-connection side chain, and part of the residues of β-(1→4) and β-(1→6) connection. Therefore, their concentration needs to be strictly restricted to prevent polysaccharides from degradation [66]. The extraction of EFPs using acid or alkali reagents is usually determined by the polysaccharide composition. For example, when EFPs contain the appreciable β-(1→6)-glucan content, acid-assisted extraction is mainly used, while when EFPs are rich in glycoprotein or a high degree of β-(1→3, 1→6)-glucan, alkaline-assisted extraction is used [67,68,69].

### 2.6. Fermentation-Assisted Extraction (FAE)

Some precious wild edible fungi are expensive and difficult to cultivate, whereas the conventional auxiliary extraction method is easy to cause an exponential increase in cost. To utilize the raw materials efficiently, the fermentation method is usually used to prepare EFPs derived from wild edible fungi. For instance, Su et al. [70] obtained bioactive exopolysaccharide (EPS) by the fermentation of *Morchella conica*. They sequentially incubated liquid medium and broth on synthetic potato dextrose agar (PDA) plates at 25 °C for one week and then transferred 10% (*v*/*v*) seed culture to the medium (pH = 6.4) and incubated at 28 °C for 5 d. After a series of impurity removal and precipitation, crude polysaccharide was obtained. He et al. [71] immersed *Pleurotus geesteranus* 5(#) in the culture medium to produce EPS, and the optimal culture conditions were determined using the orthogonal matrix method. the optimal medium (per liter) was 60.0 g maltose, 5.0 g tryptone, 1 mM NaCl, 5 mM KH_2_PO_4_, and the initial pH = 6.0 (28 °C). Under this optimal condition, the maximum EPS yield obtained was 16.97 g/L. Mao et al. [72] placed *Pleurotus geesteranus* 5(#) in PDA and then put the seed culture into a medium containing 50 mL of GP medium (0.3% peptone, 3% glucose) and cultured it at 26 °C for 4 d. They next prepared polysaccharide samples by fermenting the 4% mycelium suspension in a 5 L mixer. Likewise, a similar procedure was used to EPS from submerged cultures of *Agaricus bisporus* MJ-0811 and *Coprinus comatus*, separately [73,74].

### 2.7. Others

In addition to the extraction techniques discussed above, some popular extraction methods have recently been used, such as subcritical water extraction (SWE), pulsed electric field-assisted extraction (PEFAE), and some innovative composite extraction techniques.

**Figure 4:**
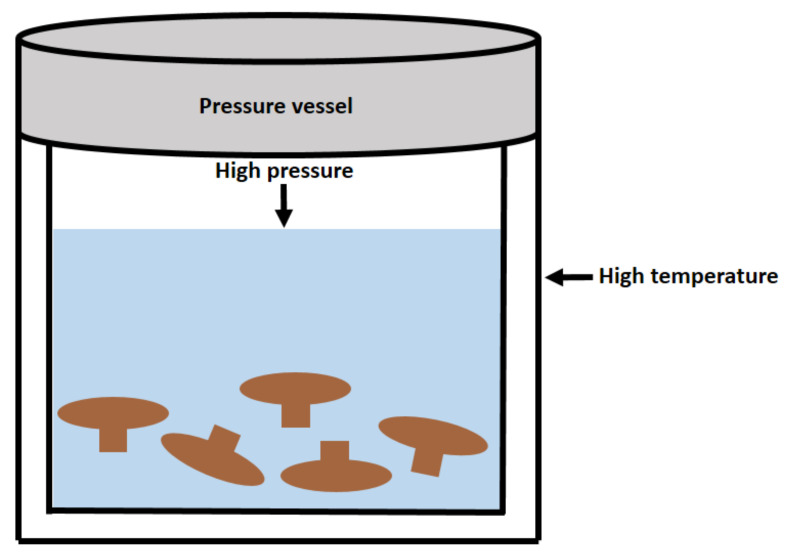
EFP extraction by SWE.

In the SWE process (Figure 4), water remains liquid at temperatures above the boiling point (100–374 °C) under sufficient pressure (1–22.1 MPa). As a result, subcritical water exhibits different properties compared with ambient pressure water at room temperature. Subcritical conditions reduce water’s dielectric constant and viscosity, enabling it to dissolve polar, moderately polar, and nonpolar compounds, including higher MW polysaccharides [17]. Additionally, subcritical water’s ionization constant increases dramatically with temperature, resembling an acidic solution and activating chemical reactions such as the hydrolysis of ether and ester bonds in polymer bonds without needing additional catalysts [33]. Seoane et al. [75] investigated the effect of temperature on *Pleurotus eryngii* polysaccharides using SWE. The oligosaccharide content was shown to increase with increasing extraction temperature, with the highest glucan content (73%) at 210 °C. At 150 °C, the glucose concentration was maximum, whereas fructose, mannitol, and trehalose reached their maximum levels at 180 °C. In addition, the glucose content was observed to increase steadily with increasing processing temperature and duration, according to a study on the impact of the SWE microenvironment on the extraction of polysaccharides from shiitake mushrooms [33,76]. Subcritical water becomes less polar as the temperature rises, while becoming more non-polar. Therefore, polysaccharides with various polarity can be extracted using a variety of processing temperatures.

**Figure 5:**
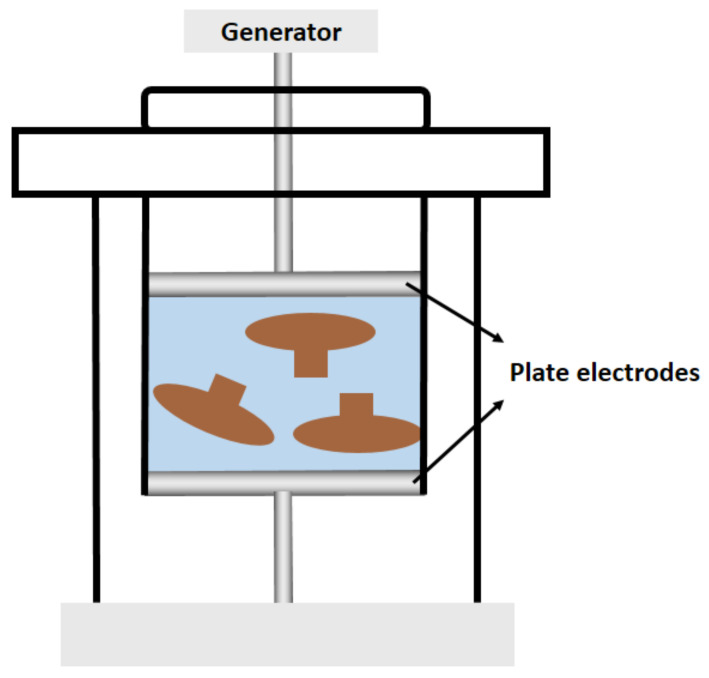
EFP extraction by PEFAE.

PEFAE is an electroporation technique that utilizes high voltage pulses (10–100 kV/cm) and durations of 100–1000 μs to induce local membrane breakdown and structural changes to enhance cell wall porosity and cell membrane permeability, thereby enhancing tissue permeability and the extraction of intercellular components (Figure 5) [30]. PEFAE has the advantages of short processing time, relatively low energy consumption (1–20 kJ/kg), and no need for thermal processing. It is suitable for the extraction of temperature-sensitive polysaccharides [77]. Liu et al. [18] extracted and separated intracellular acidic polysaccharides from *Morchella esculenta*. The optimal extraction process for obtaining acid polysaccharides was 18 kV/cm of electric field intensity, 7 for pulse number, and 27 mL/g of liquid-to-solid ratio. At this point, the crude polysaccharide yield reached the maximum value of 56.03 µg/mL. Parniakov et al. [78] compared the efficiency and stability of different extraction methods for *Agaricus bisporus* polysaccharide extraction. The results showed that the polysaccharide solution extracted by PEFAE was more straightforward and had higher colloidal stability compared with HWE and EAE.

The employment of two or more extraction methods to decrease the flaws in a single extraction process and hence increase efficiency is increasing due to the principle of complementary advantages and drawbacks. For example, Yin et al. [79] used the enzyme-microwave-ultrasonic assisted extraction method to extract polysaccharides from shiitake mushrooms. The optical extraction conditions were obtained by response surface methodology: 48 °C of enzymatic hydrolysis temperature, pH = 5.0, 440 W of microwave power, and 10 min of microwave time. Under the optimized conditions, the yield of *Lentinus edodes* polysaccharides was 9.38%, and it had significant in vitro antioxidant activity. Lin et al. investigated the preparation of polysaccharides from *Lentinus edodes* using a microwave-assisted and ethanol/(NH_4_)_2_SO_4_ solvent extraction system [80]. Under the optimal conditions of 26.0% ethanol and 19.58% ammonium sulfate, 80 °C of extraction temperature, 50 mL/g of liquid–solid ratio and 20 min of extraction time, the polysaccharide yield reached 11.2%. The microwave-assisted technique achieved higher yields and consumed less time than HWE (9.9% and 60 min) and UAE (9.8% and 30 min).

**Table 1:** Extraction method of edible fungus polysaccharides.

Extraction Method	Edible Fungus Origin	Extraction Step	Yield	Ref
Hot water extraction	*Cordyceps sinensis*	Extraction in boiling water; repeated 2 times for 2 h each time	/	[21]
	*Flammulina velutipes* mycelium	Degreasing with 95% ethanol, extraction with hot water at 95 °C for 4 h (repeated twice), rotary evaporation under reduced pressure at 50 °C, precipitation with 98% ethanol, deproteinization with cetyltrimethylammonium bromide (CTAB), decolorization by macroporous ion exchange resin, and precipitation and separation with 95% ethanol	2.2%	[22]
	*Lentinus edodes*	Extraction in hot water at 98 °C for 1 h (50 g/L, *w*/*v*), cross-flow microfiltration, and reverse osmosis (nanofiltration)	5%	[23]
	*Pleurotus florida*	Extraction in boiling water for 6 h	/	[24]
	*Sarcodon aspratus*	Pretreatment with 95% ethanol for 1.5 h and extraction with boiling water for 1.5 h (repeated 3 times)	/	[25]
	*Lentinus edodes*	Pretreatment with 95% ethanol for 24 h, extraction in boiling water twice, 1 h each time; precipitation with 95% alcohol, decolorization with H_2_O_2_, and protein removal	0.9%	[26]
	*Dictyophora indusiata*	Extraction in boiling water for 2 h (1:30, *w*/*w*) and precipitation with absolute ethanol	13.2%	[27]
	*Ganoderma applanatum*	Extraction in boiling water for 3 h and precipitation with 95% ethanol (1:3, *v*/*v*) at 4 °C for 24 h	2.14%	[28]
	*Poria cocos* Wolf	Degreasing with petroleum ether for 24 h (repeated twice), extraction in boiling water for 2 h (20:1 mL/g, *w*/*v*), precipitation with ethanol overnight (4 °C), and protein removal by Sevag method	/	[29]
Ultrasonic-assisted extraction	Medicinal Fungi	Extraction by ultrasonic (20 kHz, 130 W) at 45–50 °C for 30 min with a solid–liquid ratio of 1:3, *w*/*v*	18%	[39]
	*Flammulina velutipes* Stipe	Ultrasonic extraction (680 W) for 19.8 min at a liquid-to-solid ratio of 28 mL/g, *w*/*v*	16.20%	[40]
	*Poria cocos* Wolf	Degreasing with petroleum ether for 24 h and then extraction in a 200 W ultrasonic bath at a liquid–solid ratio of 20:1 (mL/g) for 1 h	/	[29]
	*Grifola frondosa*	Extraction by sonication (1:30 g/mL, *w*/*v*) at 50 kHz for 30 min, and then extraction in a water bath (90 °C) for 3 h and precipitation with absolute ethanol	/	[41]
	*Phellinus linteus mycelia*	Ultrasonic extraction (5.0%, *w*/*v*) at different powers (200, 400 and 600 W) in a 40 °C water bath for 30, 60, 90, 120, 150 and 180 min.	/	[42]
Microwave-assisted extraction	*Poria cocos* Wolf	Degreasing pretreatment and microwave-assisted extraction for 2 min (20:1 mL/g, *w*/*v*, 2450 MHz, 800 W)	9.95%	[29]
	Mushroom	Microwave-assisted extraction (1:30, *w*/*v*, 2455 MHz, 30 bar) and precipitation with ethanol	12.1–44.2%	[49]
	*Cordyceps gunnii mycelia*	Microwave-assisted extraction at 70 °C for 5 min (280 W) at a solid-to-liquid ratio of 1:20, *w*/*v*, repeated 3 times, followed by precipitation with ethanol at 4 °C for 12 h	30.35%	[50]
Enzyme-assisted extraction	*Poria cocos* Wolf	Degreasing with petroleum ether for 24 h, followed by extraction with 1.0% cellulase at a liquid-solid ratio of 20:1 (mL/g) for 1 h	/	[29]
	*Tricholoma mongolicum* Imai	Degreasing pretreatment, extraction with 20 g/kg cellulase in water bath for 127 min (50 g/L, *w*/*v*, pH = 4.0, 50 °C), and then precipitated with ethanol	18.96%	[8]
	*Cordyceps gunnii mycelia*	Extraction with 2% solution containing 0.5% cellulase (1:20, *w*/*v*, pH 5.0) at 55 °C for 80 min	32.50%	[50]
	*Armillaria mellea* mushroom	Extraction (30:1 mL/g, *w*/*v*) with 1.9% composite enzyme (cellulose and papain, 2:1) at 50 °C for 138 min, accompanied by 360 W ultrasonic for 20 min	40.56%	[55]
Alkali/acid-assisted extraction	*Lentinus edodes*	Extraction in boiling water, repeated 2 times for 1 h each time; treatment with 2% NaOH and 0.05% NaBH for 24 h; precipitation with 95% ethanol	4.8%	[26]
	Mushroom fruiting bodies	Degreasing with 95% ethanol (1:10, *w*/*v*); extraction in boiling water (1:20, *w*/*v*), repeated 3 times for 4 h each time; extraction with 0.5 M NaOH and a trace amount of NaBH for 3 h ×2 and 2 h ×1 at 80 °C (1:20, *w*/*v*), respectively; precipitation with 95% ethanol	1.03–16.6%,	[62]
	*Russula albonigra* (Krombh.) Fr.	Boiling in 4% NaOH for 1 h; precipitation with 1:5 (*v*/*v*) EtOH; purification by gel permeation chromatography	/	[63]
	*Phellinus linteus*	Extraction in hot water at 95 °C for 8 h, repeated twice; repeating the last step 2 times under the same conditions with 1% (NH_4_)_2_C_2_O_4_; extraction with 1.25 M NaOH/0.05% NaBH_4_ at room temperature, repeated twice for 3 h each time	9.12–19.49%	[60]
	*Pleurotus sajor-caju*	Extraction with 1% ammonium oxalate at 98 °C for 3–5 h; precipitation with ethanol; extraction with a mixture of 5% NaOH and 0.05% NaBH at 30 °C for 12–20 h	/	[64]
	*Pleurotus ostreatus*	Extraction with 3.8% HCl at 30 °C for 300 min	/	[65]
Fermentation extraction	Fermentation broth of *Morchella conica*	Precipitation with 95% ethanol after centrifugation, deproteinization, and degreasing	5.09%	[70]
	*Pleurotus geesteranus* 5(#)	Production of exopolysaccharide (EPS) in optimally defined medium (per liter) of 60.0 g maltose, 5.0 g tryptone, 1 mM NaCl, 5 mM KH_2_PO_4_, initial pH 6.0 (28 °C)	16.97 g/L	[71]
	*Pleurotus geesteranus* 5(#)	Growing in glucose peptone medium (GP) medium (0.3% peptone, 3% glucose) for 4 d (150 rpm, 26 °C); fermented in a 5 L stirrer using 4% (*v*/*v*) mycelium suspension	11.09 g/L	[72]
	*Agaricus bisporus* MJ-0811	Culture in potato dextrose agar (PDA) medium for 4 d (30 °C) and then inoculation into liquid medium (150 rpm, 4 d); precipitation with 95% ethanol at 4 °C for 24 h	2.69 g/L	[73]
	*Coprinus comatus*	Extraction in boiling water for 1 h, repeated 3 times; precipitation with 95% ethanol for 12 h	4.50–41.27%	[74]
Subcritical water extraction	*Lentinus edodes*	Subcritical water extraction at 5 MPa	11.03–14.11%	[33]
High voltage pulsed electric field-assisted extraction	*Morchella esculenta*	Extraction (27 mL/g, *w*/*v*) by high-voltage pulsed electric field (18 kV/cm electric field strength, number of pulses 7)	56.03 μg/mL	[18]
Complex extraction	*Lentinus edodes*	Extraction by microwave (440 W, 10 min) and enzyme (48 °C, pH = 5.0)	5.42%	[79]
Homogenate extraction	*Lentinus edodes*	Extraction for 66 s (30 mL/g, *w*/*v*, pH = 10)	13.2%	[81]
Vacuum extraction	*Lentinus edodes*	Extraction for 25 min (26 mL/g, *w*/*v*, 0.08 MPa, 62 °C)	4.28%	[82]
Nanoparticle extraction	*Sparassis crispa* and *Phellinus linteus*	Extraction for 2 h (20 mL/g *w*/*v*, pH = 10), emulsification with 20% nanoparticles under high pressure at 30 °C for 40 min, and then HWE at 95 °C for 30 h	54.2%	[83]

In addition, some innovative techniques for the extraction of EFPs, such as homogeneous extraction, vacuum extraction, and nanoparticle-assisted extraction, have been developed. Homogeneous extraction is a physical method that utilizes high-speed mechanical shearing, cutting, pulverization and mixing to extract biocomponents without heat and pressure. Homogeneous extraction was employed to extract polysaccharides from *Lentinus edodes* with the benefits of short processing time and mild temperature [81]. Solvent pH is the most important parameter in this method. Under the optimized conditions of pH = 10, 30 mL/g of liquid–solid ratio and 66 s of extraction time, the extraction rate reached 13.2% [81]. The crude polysaccharides obtained by homogeneous extraction exhibited stronger DPPH scavenging activity compared with conventional HWE at the same concentration. This may be due to the thermal degradation of antioxidant components in the latter method. A substantial cavitation effect caused by the generation of microbubbles during vacuum extraction enhances the solubility of biological components in the solvent medium and enables rapid extraction at low temperatures. By optimizing the conditions including 0.08 MPa of pressure, 62 °C of extraction temperature, 25 min of extraction time, and 26 mL/g of liquid–solid ratio, the maximum yield of polysaccharides reached 4.28% [82]. As for nanoparticle-assisted extraction, insoluble tungsten carbide nanoparticle powder was used for a nanoknife to extract β-glucan from *Sparassis crispa* and *Pellinus linteus* [83]. Alkaline extraction was firstly performed at pH = 10 and 20 mL/g of liquid–solid ratio for 2 h, which was followed by high-pressure emulsification with 20% nanoparticles at 30 °C for 40 min, and finally HWE treatment at 95 °C for 30 h. The yields of the two EFPs obtained by this method were 70.2% and 65.2%, respectively.

Different extraction methods affect the average MW distribution, surface morphology, and helical conformation of polysaccharide extracts, but they do not change the types of sugar rings and glycosidic bonds inside polysaccharides [84]. As the MW, structural characteristics, and monosaccharide composition of the polysaccharide fractions change with different extraction strategies, researchers can screen and choose the most appropriate extraction method based on the observed bioactivity to suit the application required for further purification steps.

## 3. Preliminary Chemical Composition of EFPs

Edible fungi contain many types of polysaccharides, including heteropolysaccharides rich in fucose, galactose, mannose, and xylose, glycogen-like glucans, and structural cell wall polysaccharides [7]. Numerous studies have demonstrated that TFP has a complex structure, with the basis for its structural heterogeneity being the molecular weight, various monosaccharide compositions, and the linkage of glycosidic links. Generally, different extraction methods will mainly affect the MW, type of glycosidic bond, length of backbone chain or branched chain, surface morphology and spiral conformation of polysaccharides, and they do not change the monosaccharide composition [45,84]. However, MW, monosaccharide composition, and glycoside bond type and connection model will significantly affect their bioactivity.

### 3.1. MW

MW has been recognized as a critical parameter reflecting the chemical properties of polysaccharides [14]. Currently, the MW of polysaccharides is typically determined using high-performance size-exclusion chromatography (HPSEC), high-performance liquid chromatography (HPLC), and high-performance gel permeation chromatography (HPGPC) [85]. HPLC is the most commonly used technique for determining the MW of polysaccharides. Zhang et al. [21] used a Sephacryl S-300HR gel column to detect the MW of the polysaccharide fraction CP-PS extracted by boiling water, the MW distribution of CP-PS was homogeneous, and its average MW was 12 kDa. Han et al. [25] used the HPSEC system to measure the homogeneity and MW of the polysaccharide from *Sarcodon aspratus*’ fruit bodies (HCP). They discovered that HCP had better homogeneity and it exclusively contained D-glucose, which had an MW of 670 kDa. Wu et al. [86] found that the MW of *Pellinus linteus* polysaccharide (PSCP) was between 22 and 1700 kDa. In the study of Wang et al. [26], the purified fraction MW values of water-extracted polysaccharide (SLNT) and alkaline-extracted polysaccharide (JLNT) from *Lentinus edodes* (SLNT1, SLNT2, JLNT1, JLNT2, and JLNT3) were 617.6, 97.57, 638.7, 273.8, and 151.3 kDa, respectively. Liao et al. [87] isolated a water-extracted polysaccharide (DP1) from the fruiting body of *Dictyophora indusiata*, and the HPGPC assay showed that the average MW of DP1 was 1132 kDa. Wang et al. [29] extracted four water-soluble polysaccharides including PCP-H, PCP-M, PCP-E, and PCP-U from *Poria cocos* Wolf using HWE, UAE, EAE, and MAE methods, respectively. Their MWs were 21.5, 21.2, 10.6, and 15.1 kDa, respectively. They found that PCP-E had the lowest MW and speculated that the enzyme might partially degrade the polysaccharide. Chen et al. [41] obtained *Grifola frondosa* polysaccharide (GFP-N) by the UAE method and measured its average MW of 12,600 kDa by HPGPC. Zhang et al. [88] used HPGPC to detect the MWs of three *Suillellus luridus* polysaccharides, and their MWs were 6.383, 8.172, and 10.710 kDa, respectively. Wang et al. [31] used gel filtration chromatography (HPGFC) to measure the MWs of ORP-1, ORP-2, and ORP-3 from *Oudemansiella radicata* mushroom, and the results showed that MW distributions of ORP- 1, ORP-2, and ORP-3 were homogeneous and their sizes were 13.921, 14.942, and 10.209 kDa, respectively. Liu et al. [32] purified *Paxillus involutus* polysaccharide with DEAE-cellulose 52 and Sephadex G-100 gels and obtained a PIP2-1 fraction with MW of 32 kDa. Xu et al. [89] used a single-factor experiment and response surface methodology to extract *Lepista nuda* polysaccharides (LNP), and two novel polysaccharides (LNP-1 and LNP-2) with MWs of 11.703 and 13.369 kDa were obtained. Generally, EFPs’ MW distribution is easily affected by its extraction method. UAE, MAE, EAE, and AAE extraction methods can reduce EFPs’ origin MW.

### 3.2. Monosaccharide Composition

There is growing evidence that the structure of polysaccharides, including the monosaccharide composition, glycosidic bond type, and conformation, can influence their biofunction. Among these, the gas chromatography (GC) and HPLC methods are typically used to assess the monosaccharide composition of EFPs by acid hydrolysis and derivatization [90]. EFPs are mainly composed of galactose (Gal), glucose (Glc) and mannose (Man) with different molar ratios. Additionally, rhamnose (Rha), arabinose (Ara), xylose (Xyl), fucose (Fuc), and ribose (Rib) may also be included (Table 2) [85]. Zhang et al. [21] used GC-MS to detect the monosaccharide composition of CP-PS polysaccharide from cultured *Cordyceps sinensis*. They found that CP-PS was a heteropolysaccharide consisting mainly of Man (38.37%), Glc (27.44%), Gal (24.25%), Xyl (5.22%), Rha (2.51%) and Ara (2.21%). Wu et al. [86] found that PSCPL polysaccharides from *Pellinus linteus* were mainly composed of Glc (37.4%), Man (2.2%), Gal (12.6%), N-acetylglucosamine (GlcNAc) (29.5%), and some unidentified monosaccharides (18.3%). Wang et al. [26] used GC-MS to analyze five purification grades of two water-extracted and alkaline-extracted crude polysaccharides (SLNT and JLNT) from *Lentinus edodes* fruiting bodies, respectively. They studied the monosaccharide composition of five purified polysaccharides, including SLNT1, SLNT2, JLNT1, JLNT2, and JLNT3. They found that the five purified polysaccharides were all composed of Glc only but had different types of glycosidic bonds, and the types of glycosidic bonds affected their antitumor activity. Liao et al. [87] used ion chromatography (IC) to analyze the monosaccharide composition of *Dictyophora indusiata* polysaccharide DP1, and they found that it was mainly composed of Glc (56.2%), Gal (14.1%) and Man (29.7%). Wang et al. [29] extracted four water-soluble polysaccharides from *Poria cocos* Wolf by HWE, UAE, EAE, and MAE methods, including PCP-H, PCP-M, PCP-E, and PCP-U. The four polysaccharides were Man, Glc, Gal, and Ara. Among them, PCP-M had the highest Man content. In terms of molar ratio, while Glc was the main monosaccharide. In addition, PCP-M also had the highest uronic acid content and carbohydrate content. Chen et al. [41] analyzed the monosaccharide composition of GFP-N from *Grifola frondosa* by the GC method. They found that GFP was mainly composed of Ara, Man, and Glc with a molar ratio of 3.79:1.00:49.70. Zhang et al. [88] extracted three polysaccharides (*Suilu*.A, *Suilu.*C and *Suilu*.S) from *Suillellus luridus* by the HWE method and used GC and efficient anion exchange chromatography (HPAEC) to determine its monosaccharide composition and uronic acid content. They found that the three polysaccharides were composed of Ara, Xyl, Man, Glc, and Gal, with the highest glucose content (41.90–49.09%) and the lowest xylose content (2.06–4.36%), but none of them contained uronic acid. Cao et al. [74] used 1-phenyl-3-methyl-5-pyrazolone (PMP) pre-column derivatization combined with HPLC to determine the monosaccharide of *Coprinus comatus* extracellular polysaccharide (EP) and intracellular polysaccharide (IP) composition. They found that EP mainly consisted of Man, Glu, Gal, Xyl, Ara, and unknown monosaccharides, while IP only contained Man, Gal, and Xyl. Wang et al. [31] obtained three kinds of *Oudemansiella radicata* polysaccharides by the HWE method, including ORP-1, ORP-2, and ORP-3. Among them, ORP-1 was mainly composed of Man, Rib, Glc, Gal, and Xyl, with a molar ratio of 2.46:2.63:3.89:3.22:2.78; ORP-2 was mainly composed of Rib, Glc, and Xyl, with a molar ratio of 2.63: 3.38:2.65; ORP-3 was mainly composed of Glc and Xyl with a molar ratio of 3.38:2.65. The *Paxillus involutus* polysaccharide prepared by Liu et al. [32] was composed of Man (2.8%), Glc (62.2%), Gal (25.4%), and Fuc (9.6%) composition. Xu et al. [89] prepared two *Lepista nuda* polysaccharides (LNP-1, LNP-2) and analyzed their monosaccharide composition. LNP-1 was mainly composed of Man, Glc, Gal, Xyl, Ara, and Fuc, whereas LNP-2 was mainly composed of Man, Glc, Gal, Ara, and Fuc. Their uronic acid contents were 5.56% and 6.80%, respectively. 

### 3.3. Glycosidic Bond Type and Backbone Structure

**Figure 6:**
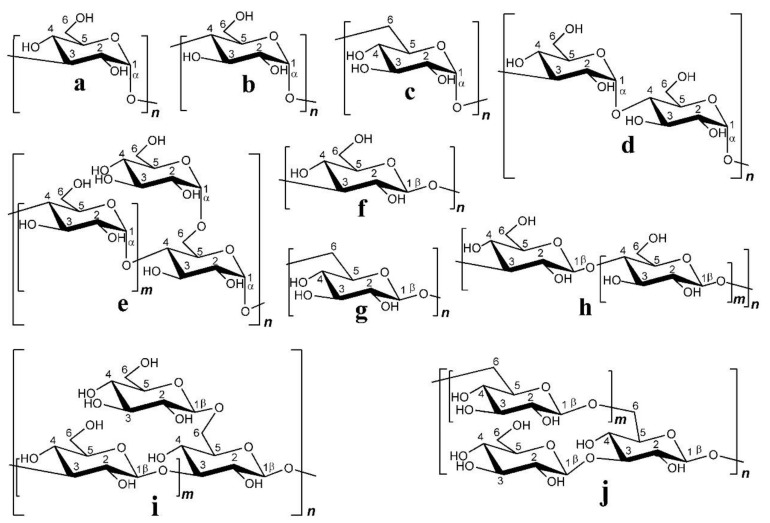
Structure of EFP glucans: (**a**) (1→3)-α-D-glucan; (**b**) (1→4)-α-D-glucan; (**c**) (1→6)-α-D-glucan; (**d**) mixed-linkage (1→3),(1→4)-α-D-glucan; (**e**) branched (1→4),(1→6)-α-D-glucan; (**f**) (1→3)-β-D-glucan; (**g**) (1→6)-β-D-glucan; (**h**) mixed-linkage (1→3),(1→4)-β-D-glucan; (**i**) branched (1→3),(1→6)-β-D-glucan; (**j**) branched (1→6),(1→3)-β-D-glucan. Reprinted with permission from Ref. [93]. Copyright 2022, Elsevier.

**Figure 7:**
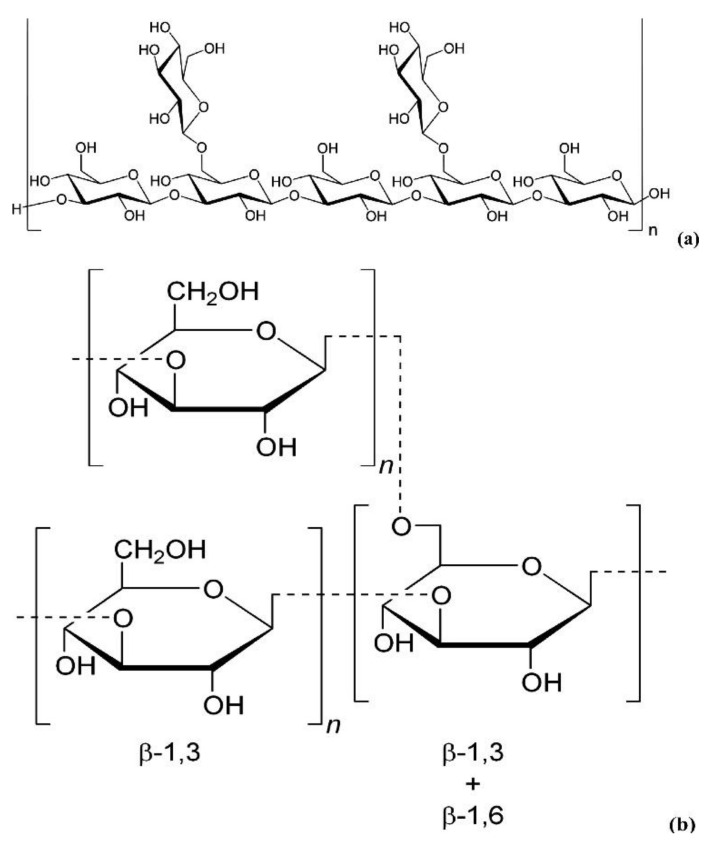
Structural diagram for (**a**) lentinan and (**b**) schizophyllan. Reprinted with permission from Ref. [5]. Copyright 2022, Elsevier.

As one of the most important active components in edible fungi, EFPs are composed of various neutral sugars or uronic acids through the complex polymerization of glycosidic bonds [91]. The EFPS structure mainly includes glucan and heterosecan [92,93]. Glucan refers to D-glucose polymers with different MWs and spatial configurations (Figure 6), whereas the heterosecan is composed of a variety of monosacoly according to different molar ratios. The backbone and branches of polysaccharides are usually elucidated by periodate oxidation, Smith degradation, and methylation assays [89]. For instance, methylation analysis revealed that water-soluble polysaccharides from mushroom *Lepista nuda* (AAP) prepared by the HWE method mainly consisted of 1,4-linked-Glc p, 1,4,6-linked-Glc p, terminal Glc p, 1,4 -linked-Man p, 1,2, 6-linked-Man p, and terminal Arap p [94]. Decha used Fourier transform infrared spectroscopy (FT-IR) to analyze the type of glycosidic bonds of polysaccharides extracted from *Pleurotus sajor-caju* and found that the polysaccharides contained β-(1→3) glucans and mannans [64]. Biscaia et al. [95] isolated a heteropolysaccharide from *Pleurotus eryngii* by cold water extraction. NMR spectroscopy, monosaccharide composition, and methylation analyses revealed that it has the main chain of (1→6)-linked α-d-galactopyranosyl and 3-O-methyl-α-d-galactopyranosyl residues, and both partially substituted at OH-2 by a β-d-Manp (MG-Pe) single-unit. Liu et al. analyzed the glycosidic bond types of *Agaricus bisporus* polysaccharide (ABP) by FT-IR and periodic acid oxidation, and they found that ABP Ia was an α-pyran polysaccharide consisting of 1→2, 1→4, and the possible 1→3 glycosidic bonds [96]. The composition of the glycosidic linkages in EFPs is greatly influenced by the various extraction techniques. For instance, Liu et al. used fractional ethanol precipitation to extract three polysaccharides (PUP) fractions from *Polyporus umbellatus* [97]. The backbones were all composed of α-Man p and β-Man p despite the differences in their monosaccharide compositions and MWs. Li et al. [98] extracted PUP *Polyporus umbellatus* (pers.) with boiling water. They found that its backbone mainly had a β-D-linked Glcp structure. In contrast, the backbone of the PUP from *Polyporus umbellatus* sclerotia by boiling water extraction was composed of (1→6, 1→3)-β-linked Glcp and had a branching degree of 22.8% and a small amount of uronic acid [99]. In addition, the backbone of another PUP extracted by HWE consisted of (1→6)-β-D-glucopyranosyl (Glcp), every second of which was substituted at O-3 by side chains composed of terminal β-D-Glcp, (1→3)-β-D-Glcp, (1→3)-β-D-GlcpA, (1→4)-β-D-Glcp and (1→4)-β-D-GlcpA units [100]. He et al. [101] extracted PUP from *Polyporus umbellatus* by HWE and found that the main repeating unit of its structure was identified as an α-(1→6)-D-galactopyranan backbone with the substitution of terminal a-galactopyranosyl residues at 0–2 for two out of every three main chain galactose residues. Yan et al. [102] purified 3-O-methylated heterogalactose (WPEP-N-b, 21.4 kDa) from *Pleurotus eryngii* fruiting bodies and found that the main chain of WPEP-N-b was composed of α-1,6-linked D-Galp and 3-O-MeD-Galp branched at O-2 with single t-β-D-Manp as the major side chain. Beta-1, 6-D-Glcp residues were present as minor components either in the side chains or backbone. Chen et al. [103] reported that the possible structure of the primary glycosidic linkage in (EP-1) from the purified fractions of *Pleurotus eryngii* polysaccharide was β-(1→3)-glucan glycosidic linkage, which is branched at O-6 by α-D-glucose. It should be noted that EFPs’ structure is specific, and their bioactivity highly depends on structure. EFPs with excellent features mainly belong to β-glucan but a small part of the α-configuration, and they also contain some heterosecans [5,104]. For example, some of the mushroom β-glucans, such as lentinan from *Lentinula edodes* and schizophyllan from *Schizophyllum commune* have been recognized as immunoceuticals in countries such as China, Japan, and Korea [83]. The structural properties and bioactivities of mushroom β-glucan differ depending on the source. Figure 7 shows the structure of two distinct mushroom β-glucans (lentinan and schizophyllan) [5]. In addition, the main chain of EFPs usually contains (1→3), (1→4), (1→6), and mixed glycoside bonds.

## 4. In Vitro Bioactivity of EFPs

As a popular bioactive polysaccharide, EFPs have been reported to exhibit excellent performance in various in vitro activity evaluation models (Table 3).

### 4.1. Antineoplastic Activity

**Figure 8:**
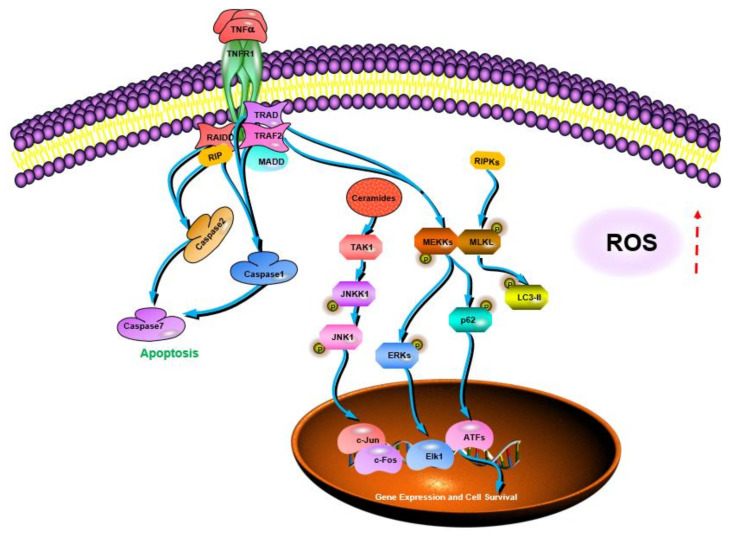
Antineoplastic signaling mechanism of edible fungal polysaccharides in an in vitro cell model.

**Figure 9:**
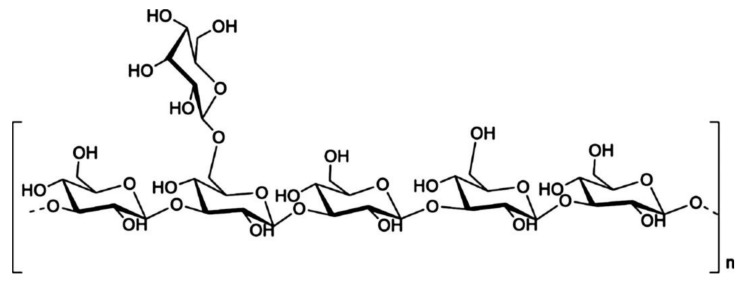
A schematic diagram of β-glucan structure in *Poria cocos* [112].

Cancer is a malignant disease characterized by a group of uncontrolled cell growth with a tendency to invade adjacent parts of the body, transforming normal cells into malignant cells. Although the development of treatment methods or technologies, including surgery, radiotherapy, and chemotherapy, have improved the survival rate of patients with primary malignant tumors, the toxic side effects and high recurrence rate of anticancer therapy have prompted the urgent need to explore new cancer therapies. Recent studies have shown that EFPs can exert anticancer activity by activating T lymphocytes, macrophages, and NK cells (Figure 8). It has been reported that EFPs from *Gleoestereum incarnatum* could interact and bind to tumor cell surface receptors to induce tumor cell apoptosis [31]. Due to the specific configuration and high affinity for cellular receptors, polysaccharides extracted from several species of *Lentinus edodes* showed significant inhibitory activity against tumor cells. Wang et al. [26] investigated the in vitro antitumor effects of five *Lentinus edodes* polysaccharides (SLNT1, SLNT2, JLNT1, JLNT2, and JLNT3) on mouse hepatoma H22 cells and human hepatoma cell lines including HepG2 and SMMC-7721. They found that all five EFPs could significantly inhibit tumor cell growth in a concentration-dependent manner. Notably, at the highest experimental concentration (800 μg/mL), SLNT1 and JLNT1 had the strongest anti-proliferation activity on mouse hepatoma H22 cells, and the inhibition rates were 61.48% and 62.32%, respectively, reflecting their selectivity for tumor cells inhibition. Li et al. [105] studied the inhibitory activity of SLNT on human breast cancer. They found that SLNT mainly inhibited the proliferation of MCF-7 cells in a dose-dependent manner, with the highest inhibition rate reaching 45.46%. In addition, SLNT could trigger mitochondrial apoptosis by activating caspase-7 and simultaneously activate autophagy in MCF-7 cells, thereby accelerating MCF-7 cell apoptosis. *Russula griseocarnosa* polysaccharide (PRG) has also been reported to show significant antitumor activity. After incubation with PRG, the cell viability of cervical cancer cell lines, including Hela and Siha, decreased in a dose-dependent manner [79]. Pan et al. [106] found that *Ganoderma lucidum* polysaccharide (GLP) could activate the MAPK/ERK pathway and then elevate the levels of LC3-II and p62, thereby promoting the accumulation of autophagosomes and leading to autophagy and apoptosis in rectal cancer cell lines of HT-29 and HCT116. Li et al. [107] analyzed the anticancer activities of *Russula virescens* polysaccharides (RVP-1 and RVP-2) and found that both RVP-1 and RVP-2 could significantly inhibit human breast cancer MCF-7 cell proliferation. According to reported studies, many polysaccharides with anticancer potential contain four to six different monosaccharides, including mannose, xylose, fucose, rhamnose, and ribose [108]. Some polysaccharides containing uronic acid and protein or sulfated form also exhibit excellent antitumor activity. Pawlikowska et al. [109] investigated the in vitro antitumor activity of protein-bound polysaccharides (PBPs) from *Coriolus versicolor*. The results indicated that PBPs could dose-dependently mediate necroptosis in breast cancer and melanoma cells by activating the RIPK1/RIPK3/MLKL pathway. Moreover, PBP also induced the activation of the TNF-α/TNFR1 pathway in breast cancer cells, inhibited the expression of TNF-α in pigment melanoma cells, and triggered the production of ROS, thus inducing intracellular necroptosis. Similar results were obtained in their other study [110]. They found that PBP could cause caspase-independent cell death, which was regulated by receptor-interacting protein-1 (RIP-1) and ROS and modified by melanin. Li et al. [111] compared the in vitro anticancer activities of two *Lentinus edodes* polysaccharides (LPS and HPLPS) obtained by HWE and ultrahigh-pressure (UHP) extraction methods. The results showed that both polysaccharides showed anti-proliferative activities on HepG2 and HeLa cells, but the inhibitory effect of HPLPS was stronger, which might be related to the increase in homogeneous polysaccharide composition in Lentinus edodes by UHP treatment. In addition to polysaccharide composition, the structure also affects the antineoplastic activity of EFPs. The structure of a polysaccharide from *Poria cocos* with strong antineoplastic activity is shown in Figure 9, which is mainly composed of β-glucan, with a β-(1→3)-linked glucose backbone and β-(1→6)-linked glucose side chains [112].

### 4.2. Immunomodulatory Activity

**Figure 10:**
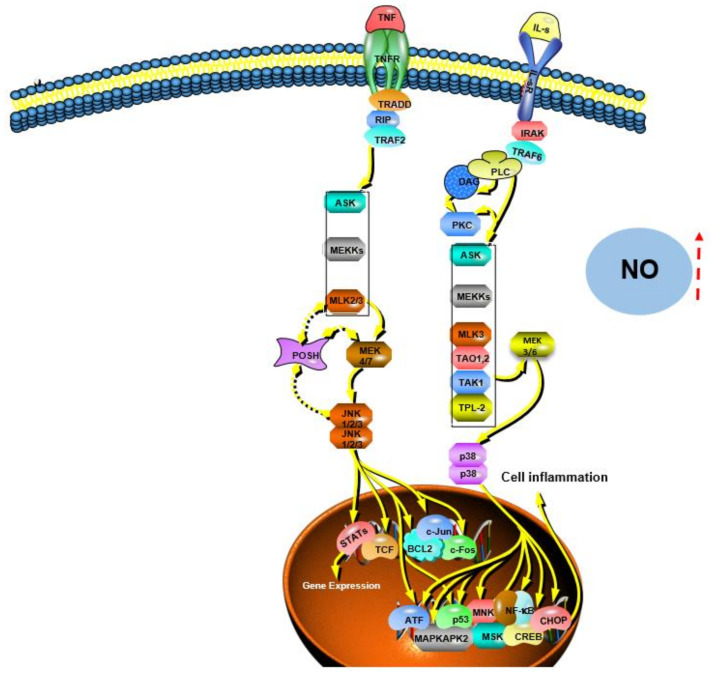
Immunomodulatory signaling mechanism of edible fungal polysaccharides in an in vitro cell model.

**Figure 11:**
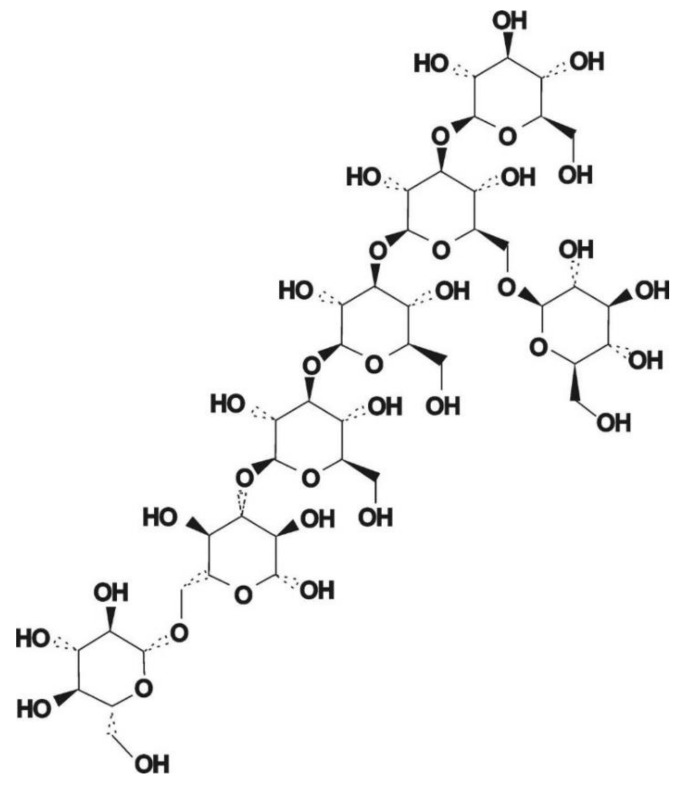
Lentinan. Reprinted with permission from Ref. [118]. Copyright 2022, Elsevier.

Immune stimulation is considered one of the critical strategies for improving the body’s defenses in older people and cancer patients [113]. Extensive experimental evidence suggests that EFPs can enhance the immune system by stimulating natural killer (NK) cells, T cells, B cells, and macrophage-dependent immune responses [114]. Based on their ability to function and cytokine profile, T cells were characterized as CD4 Th1 cells or CD8 Th2 cells. Th2 cells secrete IL-4 and IL-10 and activate humoral immunity, whereas Th1 cells secrete interferon (IFN) and promote cell-mediated immunity. The balance between these two subtypes is crucial for the immune response since the cytokines produced by these two subtypes have various immunological functions. Phagocytosis is the first step in the response of macrophages to pathogens when a pathological stimulus or injury stimulates the body. In addition, macrophages can defend against pathogen invasion by secreting pro-inflammatory cytokines such as TNF-α and IL-1 [115] and releasing cytotoxic and inflammatory molecules such as NO and ROS [116]. EFPs are an important immunomodulatory member in mushrooms and an essential source of natural immunomodulators (Figure 10) [117]. A typical β-glucan with immunostimulatory activity and one of the longest used in treatment is lentinan from *Lentinula edodes* (Shiitake) (Figure 11) [118]. Zhang et al. [21] reported that CP-PS enhanced the SOD activity, reduced the MDA concentration in the serum of immunosuppressed mice, and regulated the secretion of cytokines, including IL-4, IL-5, and IL-17, to enhance the body’s immune response. Yin et al. [22] isolated a water-soluble glucan FVP2C from the hot water extract of *Flammulina velutipes* mycelium and found that FVP2C could significantly promote macrophages in vitro NO production, IL-1 production, and TNF-α secretion. Dong et al. [119] studied the immunostimulatory activity of PFIO in RAW264.7 macrophages. They found that PFIO could promote NO/ROS production, TNF-α secretion, and phagocytic uptake in macrophages as well as promote the proliferation and division of mouse splenocytes. Furthermore, it could effectively promote macrophage activation through MAPK and NF-κB signaling pathways, potentially modulating immune responses. Su et al. [70] investigated the immunomodulatory activity of water-soluble *Morchella conica* polysaccharide (MCP) extracted and isolated from the fermentation broth of *Morchella conica* using an in vitro cell model. They found that MCP could regulate NO production in macrophages and modulate the innate immune response within a specific concentration range. In addition, it could promote the proliferation of spleen cells, thereby exerting a strong in vitro immunomodulatory effect. Wu et al. [86] found that the polysaccharide fraction of *Pellinus linteus* (PSCPL) could significantly protect premonocytes (THP-1) from LPS-induced toxicity compared to the aqueous and alcoholic extracts. Its mechanism may be associated with the inhibition of MyD88-dependent and MAPK signaling pathways, thereby inhibiting the formation of intracellular ROS, production of cytokines (TNF-α, IL-1α, IL-1β, and IL-4), VCAM-1 expression, JNK and p38 activation and phosphorylation, and inhibition of NF-κB activation. Yang et al. [120] found that *Coriolus versicolor* mushroom polysaccharides (CVP) could target and bind potential immune receptors including membrane Ig and TLR-4 to induce B cell activation. In addition, CVP could activate mouse B cell immunity through MAPK and NF-κB signaling pathways.

### 4.3. Antioxidant Activity

Reactive oxygen species (ROS) are independent molecules containing at least one oxygen atom and one or more unpaired electrons, such as superoxide anion radical (O_2_^−^), hydroxyl radical (OH^−^), hydroperoxide radical radicals, singlet oxygen, and free nitrogen radicals. Among them, OH^−^ is considered to be the most toxic and reactive. It can react with almost all biological macromolecules in living cells and cause damage to adjacent biological molecules, which is the main cause of peroxidative damage in biological organisms [121]. Excessive free radicals and ROSs can cause damage to DNA, RNA, membrane lipids, proteins, and other substances, leading to various diseases [122,123]. The antioxidant mechanism of EFPs has been extensively studied, and one of the potential mechanisms is that they cause the anomeric hydrogen atoms to interact with free radicals, ending the free radical chain reaction [124,125]. Wang et al. [29] evaluated the in vitro antioxidant capacity of *Poria cocos* Wolf polysaccharides (PCPs) obtained by four different extraction methods. The results showed that the antioxidant activities of the four PCPs were enhanced with the concentration increase. Among them, the reduction capability of PCP-M (obtained by MAE) and PCP-E (obtained by EAE) to Fe^3+^ was always higher at the tested concentrations. The Fe^2+^ chelation activity, DPPH, and OH^−^ scavenging activities of PCP-M were significantly higher than those of other polysaccharides. Mao et al. [73] found that exopolysaccharides from *Agaricus bisporus* (EPS) had high in vitro antioxidant activity. At the highest tested concentration (1.2 mg/mL), the scavenging rates of EPS against OH^−^ and O_2_^−^ could reach 76% and 82.45%, respectively. Similar results were obtained by Cao et al. [74]. In addition, they also found that the in vitro antioxidant capacity of crude polysaccharides was stronger than that of the purified polysaccharide. Wang et al. [31] evaluated the in vitro antioxidant activity of *Oudemansiella radicata* polysaccharides (ORPs). Among them, the DPPH scavenging activity of ORPs increased with concentration. At the highest tested concentration (4.0 mg/mL), the scavenging rates of ORP-1, ORP-2, and ORP-3 were 83.4%, 73.2%, and 59.8%, respectively. The antioxidant activity of polysaccharides is usually affected by many factors, such as their MW, water solubility, uronic acid content, monosaccharide composition, glucosidic bond type, and extraction method. Generally, high-MW polysaccharides have better biological properties and antitumor activity [126], and this may be because high-MW (MW > 90 kDa) polysaccharides are prone to form triple helix structures [33]. Low-MW polysaccharides are also preferred in certain applications, especially for therapeutic use, because their lower MW readily crosses biological barriers and enhances bioavailability [77,127,128]. In addition, monosaccharide composition also greatly affects the antioxidant activity of polysaccharides [124,129]. The high proportion of glucose and galactose in *Dendrobium nobile* Lindl polysaccharides (ORP-1) may be critical for its antioxidant activity [130]. At 4.0 mg/mL, the ABTS scavenging activity (78.3%), OH^−^ scavenging activity (67.4%), O_2_^−^ scavenging activity (13.4%), and ferrous ion chelating activity (51.2%) of ORP-1 is the highest. Another study revealed that HWE-extracted *Russula griseocarnosa* and *Paxillus involutus* polysaccharides had higher superoxide radical scavenging activity compared with other polysaccharides [32,79]. Compared with the purified polysaccharide fraction, a crude polysaccharide from *Lepista nuda* exhibited stronger O_2_^−^ and DPPH radical scavenging activity and ferrous ion chelating activity [89]. The higher antioxidant activity of crude polysaccharides may be due to the content of impure fractions or the synergistic antioxidant effect of complex polysaccharides [131].

### 4.4. Glycosidase Inhibitory Activity

Diabetes mellitus is a chronic disease characterized by hyperglycemia, which is associated with abnormal dietary metabolism and leading to chronic complications [132]. Dietary carbohydrates are first broken down into oligosaccharide fragments by α-amylase secreted by the salivary glands and pancreas, and then, they are digested into monosaccharides by α-glucosidase secreted by the small intestine. Finally, the glucose produced enters the blood and increases blood glucose levels [133,134,135]. Inhibiting digestive enzymes can prevent the rise in blood glucose and help manage the development of diabetes [136,137]. Cao et al. [74] detected the α-amylase inhibitory activity of four *Coprinus comatus* polysaccharides. When the concentration was 2–10 mg/mL, all four polysaccharides significantly inhibited α-amylase activity. In particular, the inhibitory effect of ICPS was considerably higher than that of the other three polysaccharides, and its inhibition rate at the highest concentration reached 87.15%. Li et al. [107] found that *Russula virescens* polysaccharides, including RVP-1 and RVP-2, had the potential to inhibit α-glucosidase and α-amylase activities and showed a specific concentration dependence. At the highest concentration (3.2 mg/mL), RVP-1 had a better inhibitory effect on α-glucosidase (77.59%), while RVP-2 had a more significant inhibitory effect on α-amylase (77.59%). It has been reported that the complex structure and indigestible characteristics of polysaccharides (high MW) may interfere with the activity of digestive enzymes and slow down glucose uptake [138]. The inhibitory effects of RVP-1 and RVP-2 on α-glucosidase and α-amylase may be related to the structural configuration and MW distribution. In addition to the role of digestive enzyme activity, the increased production of advanced glycation end products (AGEs) is also one of the major factors in diabetes and diabetic complications. Shen et al. [139] found that the alkali-extracted polysaccharides (AAPs) and acid-hydrolyzed polysaccharides (AAPs-F) from *Auricularia auricula-judae* could significantly and dose-dependently inhibit the formation of AGEs caused by short-term and long-term glycosylation. In addition, the glucose uptake of HepG2 cells increased by 24.4% after incubation with AAPs-F. Cao et al. [140] used spectroscopic techniques to study the interaction of *Lentinus edodes* mycelia polysaccharide (LMP) with α-glucosidase and its inhibitory effect on the formation of AGEs. The results indicated that LMP had a reversible inhibitory impact on α-glucosidase activity in a mixed-type manner. When the LMP concentration was 2.7 mM, the inhibition rate was 34.38%. It has been reported that the α-glucosidase inhibitory activity of polysaccharides is mainly related to the combined effect of several factors such as MW and monosaccharide composition [141]. The inhibitory effect of LMP on α-glucosidase may be related to its monosaccharide composition and MW. Meanwhile, LMP could inhibit the formation of AGEs. Compared with the 40 mM glucose-treated group, 0.05 mM LMP treatment for 48 h increased cell viability from 70.17% to 91.14%, and ROS production decreased from 3.33-fold to 1.21-fold. LMP also exerted a hypoglycemic effect by inhibiting the activation of MAPK signaling pathway in a model of high glucose-induced oxidative damage in human umbilical vein endothelial cells (HUVECs).

**Table 3:** Bioactivity and mechanism of edible fungus polysaccharides.

Bioactivity	Edible Fungus Origin	Regulatory Mechanism	Ref
Antioxidant activity	*Poria cocos* Wolf	Favorable in vitro Fe^2+^ chelation, DPPH scavenging, and hydroxyl radical scavenging activities	[29]
	*Agaricus bisporus*	Strong in vitro scavenging activity of hydroxyl radicals (OH^−^) and superoxide radicals (O_2_^−^)	[73]
	*Coprinus comatus*	Excellent in vitro Fe^2+^ chelation, 1,1-diphenyl-2-picrylhydrazyl (DPPH), and OH^−^ scavenging activities	[74]
	*Oudemansiella radicata*	Strong DPPH, 2,2-azino-bis (3-ethylbenzothiazoline-6-sulphonic acid) (ABTS), OH^−^, ·O_2_^−^ scavenging activity, and Fe^2+^ chelation activity	[31]
	*Russula griseocarnosa*	Strong DPPH, ABTS, OH^−^, and ·O_2_^−^ scavenging activities	[79]
	*Paxillus involutus*	Significant hydroxyl, DPPH, ABTS, and ·O_2_^−^ radical scavenging activities	[32]
	*Lepista nuda*	Strong capability to chelate iron ions, scavenge DPPH and ·O_2_^−^ radicals	[89]
Immunostimulatory activity	*Cordyceps sinensis*	Enhancing SOD activity, reducing MDA concentration, and regulating the secretion of cytokines IL-4, IL-5, and IL-17 in serum	[21]
	*Flammulina velutipes* mycelium	Significantly promoting NO production, interleukin-1 (IL-1) production, and tumor necrosis factor-α (TNF-α) secretion in macrophages	[22]
	*Inonotus obliquus*	Promoting NO/ROS production, TNF-α secretion, and phagocytic uptake in macrophages; promoting the proliferation and complexation of mouse splenocytes; effectively promoting macrophage activation through MAPK and NF-κB signaling pathways	[119]
	*Morchella conica*	Regulating NO production in macrophages, modulating innate immune responses within a specific concentration range, and promoting splenocyte proliferation	[70]
	*Phellinus linteus*	Inhibiting MyD88-dependent and MAPK signaling pathways, resulting in intracellular inhibition of ROS formation, cytokine (TNF-α, IL-1α, IL-1β, and IL-4) production, VCAM-1 expression, JNK, and p38 activation and phosphorylation, and NF-κB activation	[86]
	*Coriolus versicolor* mushroom	Directly binding mIg with repetitive epitopes, allowing efficient cross-linking of mIg on the B cell surface and driving B cell proliferation and Ig production, interacting with TLR4 to stimulate B cell activation	[120]
	*Cordyceps taii*	Increasing thymus weight and enhancing immune function of pancreatic β cells in STZ-induced diabetic mice	[142]
Antineoplastic activity	*Lentinus edodes*	Directly inhibiting the in vitro proliferation of tumor cells H22, HepG2, and SMMC-7721; significantly inhibiting the growth of tumors in mice and greatly increasing the levels of IL-2 and TNF-α in serum; inducing tumor cell apoptosis	[26]
	*Lentinus edodes*	Inhibiting the proliferation of MCF-7 cells; triggering the mitochondrial apoptosis pathway by activating caspase-7 and activating autophagy in MCF-7 cells, thereby accelerating cell apoptosis	[105]
	*Russula griseocarnosa*	Inhibiting the proliferation of Hela and Siha cervical cancer cells	[79]
	*Coriolus versicolor*	Mediating the necroptosis of cancer cells by activating the RIPK1/RIPK3/MLKL pathway; inducing the activation of the TNF-α/TNFR1 pathway in breast cancer cells; inhibiting the expression of TNF-α in melanoma cells and triggering the production of ROS	[106]
Anti-obesity activity	*Pleurotus eryngii*	Inhibiting lipid absorption and increasing mesenteric fat, reducing body weight, promoting LDLR expression, and enhancing serum LDL cholesterol uptake	[143]
	*Dictyophora indusiata*	Reversing HFD-induced changes in obesity-related parameters, lowering body weight, and reducing the degree of lipid accumulation; decreasing the expression of lipid genes (PPAR-γ, C/EBPα, and SREBP-1c)	[144]
	*Pleurotus geesteranus* 5(#)	Lowering total cholesterol, triacylglycerol, LDL cholesterol, HDL cholesterol, and atherogenic index	[72]
	*Ganoderma applanatum*	Slowing the rate of food intake in obese rats; significantly reducing TC, TG, LDL-C levels, and atherosclerosis index	[28]
	*Cordyceps taii*	Regulating lipid metabolism parameters (serum levels of TC, TG, LDL-C, and HDL-C) in diabetic mice and effectively improving lipid disorders	[142]
Hypoglycemic activity	*Pleurotus geesteranus* 5(#)	Improving pancreatic β-cell damage, promoting insulin synthesis, and lowering blood glucose	[72]
	*Grifola frondosa*	Decreasing fasting blood glucose (FBG) levels; regulating blood biochemical parameter levels and inflammatory responses in the liver and kidneys; improving glucose consumption and alleviating the insulin resistance (IR) by increasing IRS1/PI3K/GLUT4 gene/protein expression and decreasing JNK1/2 gene/protein expression	[41]
	*Suillellus luridus*	Increasing serum insulin levels, enhancing antioxidant enzyme (SOD, CAT, and GPx) activity in the liver and kidneys, and decreasing MDA levels	[88]
	*Cordyceps taii*	Reducing FBG level and IR, increasing insulin level, significantly improving islet structure damage, and promoting β-cell proliferation	[142]
	*Coprinus comatus*	Significant in vitro alpha-amylase inhibitory activity	[74]
	Mushroom fruiting bodies	Activating autophagy to reduce IR and fat deposition; effectively lowering blood glucose and protecting islets from damage caused by hyperglycemia	[62]
Intestinal homeostasis-regulating activity	*Dictyophora indusiata*	Reversing gut dysbiosis and increasing beneficial flora, including *Lactobacillus* and *Ruminococaceae*, decreasing endotoxemia by decreasing lipopolysaccharides (LPSs) and pro-inflammatory cytokines levels, such as TNF-α, IL-6, and interleukin-1β (IL-1β), and increasing the expression of claudin-1, occludin, and zonula occludens-1	[27]
	*Pleurotus eryngii*	Altering the gut environment to increase the abundance of short-chain fatty acid (SCFA)-producing bacteria (*Anaerostipes* and *Closterridium*) and decrease the abundance of *Roseburia*	[143]
	*Dictyophora indusiata*	Restoring the altered bacterial flora caused by the HED diet and increasing bacterial diversity; decreasing the Firmicutes to Bacteroidetes ratio	[144]
	*Ganoderma lucidum and Poria cocos*	Remodeling gut microbiota composition, reducing species richness, reducing Firmicutes/Bacteroidetes ratio, and significantly promoting lactic acid-producing (LAP) and SCFA-producing bacterial growth	[145]
	*Grifola frondosa*	Increasing the abundance of Bacteroidetes in the gut while decreasing the numbers of Firmicutes and Proteus; increasing the relative abundance of SCFA-producing bacteria, including Alloprevotella and Blautia	[41]
Anti-inflammatory activity	*Dictyophora indusiata*	Reducing pro-inflammatory cytokine levels (TNF-α, IL-1β, and IL-6) in adipose and liver tissue while enhancing anti-inflammatory cytokines ((IL-4 and IL-10) expression	[144]
	*Inonotus obliquus*	Inhibiting the NF-κB, COX-2, and iNOS signaling pathways	[146]
	*Agaricus bisporus*	Inhibiting the expression of pro-inflammatory genes, including IL-1β and COX-2	[147]
Anti-aging activity	*Agaricus bisporus*	Strong OH- and DPPH free radical scavenging and iron ion chelation capability, significantly improving D-galactose-induced liver damage, kidney damage, and early dysregulation of lipid metabolism	[148]
	*Flammulina velutipes*	Strong DPPH, OH- radical scavenging, and Fe^2+^ chelating activity; enhancing antioxidant enzyme (SOD, GSH-Px, CAT, and T-AOC) activities; reducing lipid peroxidation (MDA), and improving inflammatory response (reducing Ach E and NOS activities)	[149]

## 5. In Vivo Bioactivity of TFPs

**Figure 12:**
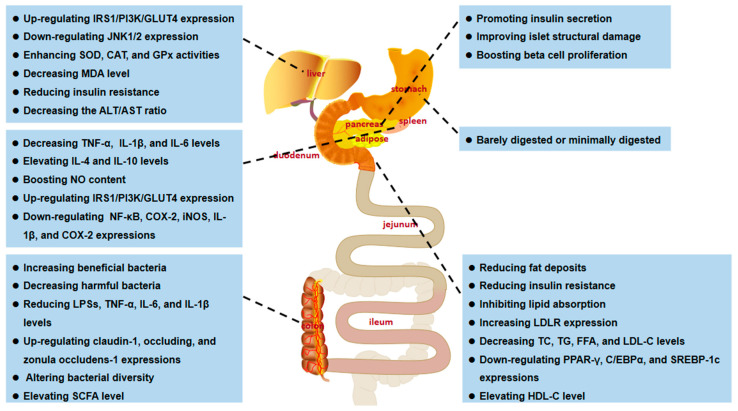
A potential mechanism by which edible fungal polysaccharides exert their *in vivo* bioactivity.

In addition to the presented in vitro bioactivities, EFPs also exhibited excellent *in vivo* bioactivities (Table 3), and their potential regulatory mechanism is shown in Figure 12.

### 5.1. Antidiabetic Activity

Diabetes mellitus (DM) is a chronic metabolic disease caused by a series of glucose metabolism dysfunctions in the body, resulting in the continuous elevation of blood glucose [150]. If this trend cannot be controlled, it further leads to many long-term microvascular and microvascular complications, such as retinopathy, cardiomyopathy, neuropathy, and nephropathy [151]. It is well known that impaired glucose tolerance, disturbance of gut microbiota, insulin resistance (IR), and increased fasting blood glucose (FBG) are the initial factors in the formation of DM [151,152]. Therefore, targeted therapies that directly impact them may have important implications for managing DM. Mao et al. [72] found that exopolysaccharides from *Pleurotus geesteranus* 5# (EPS) had a specific hypoglycemic effect. EPS treatment repaired pancreatic β-cell damage caused by the administration of streptozotocin (STZ) and acted as an insulin-like factor to promote insulin synthesis, thereby reducing plasma glucose levels (17.1%). Chen et al. [41] explored the antidiabetic activity of *Grifola frondosa* polysaccharide (GFP-N) obtained by ultrasound-assisted extraction. They found that high-dose GFP-N treatment (150 mg/kg) increased the body weight of mice for a short time, but low-dose GFP-N (75 mg/kg) treatment decreased high FBG levels more rapidly. GFP-N-fed mice could maintain normal levels of biochemical blood parameters, and high-dose GFP-N treatment significantly improved inflammatory responses in the liver and kidneys. In addition, GFP-N could improve glucose metabolism and alleviate IR by upregulating the expression of the IRS1/PI3K/GLUT4 pathway and reducing the expression of the JNK1/2 gene/protein, thereby enhancing glucose uptake in the liver. Zhang et al. [88] extracted three polysaccharides from the fruiting bodies, caps and petioles of *Suillellus luridus* (*Suilu*. A, *Suilu*. C, and *Suilu*. S), and their antidiabetic effects were evaluated. The results indicated that these polysaccharides all exhibited potential antidiabetic effects. They effectively improved weight loss, increased serum insulin levels, significantly enhanced antioxidant enzyme (SOD, CAT, and GPx) activities in the liver and kidneys, and decreased MDA levels in STZ-induced diabetic mice. Zhang et al. [153] reported that polysaccharides from *Pleurotus eryngii* SI-04 (EPS1) exhibited a strong antidiabetic activity in mice with STZ-induced diabetic nephropathy. Liu et al. [142] evaluated the hypoglycemic efficacy of polysaccharides (CTP) from *Cordyceps taii* in STZ-induced diabetic mice. They found that CTP treatment also promoted weight gain in DM mice dose-dependently. Meanwhile, CTP treatment could decrease IR and FBG levels and increase insulin levels. Furthermore, it significantly improved the damage to the islet structure and promoted β-cell proliferation. Yang et al. [62] extracted six kinds of alkali-soluble polysaccharides, neutral polysaccharides, and acidic polysaccharides from different mushroom fruiting bodies. In addition, they compared the effects of these polysaccharides on improving insulin resistance using high-fatty acid and glucose-induced hepatocytes and using a db/db diabetes mouse model. They found that all polysaccharide samples could reduce IR and fat deposition by activating autophagy, effectively lower blood glucose, and protect pancreatic islets from damage caused by high blood glucose.

### 5.2. Anti-Obesity Activity

Obesity is a complex disease characterized by the excessive accumulation of fat. Long-term obesity increases the risk of other conditions such as diabetes, hypertension, and hyperlipidemia [154]. Indigestible EFPs are commonly fermented by microorganisms in the gut as prebiotics (or dietary fibers), with positive effects on host health. Several studies have indicated that prebiotics or dietary fiber intake can improve diet-induced obesity [155]. Many EFPs contain β-glucan [156], and some β-glucans have been shown to reduce the risk of obesity [157]. Nakahara et al. [143] investigated the effect of *Pleurotus eryngii* polysaccharides (PEP) on body weight and lipid metabolic profile in mice fed a high-fat diet (HFD). The results showed that the dietary supplementation of PEP inhibited lipid absorption in obese mice, inhibiting increased body weight and mesenteric fat gain. In addition, the supplementation of PEP was beneficial in promoting the expression of LDL receptor (LDLR), thereby enhancing the uptake of LDL-C in the blood and reducing the level of serum TC. Kanwal et al. [144] studied the regulatory effect of *Dictyophora indusiata* polysaccharide (DIP) on HFD-induced obese mice. It was found that DIP diet intervention could effectively reverse HFD-induced changes in obesity-related parameters (such as ALT, AST, TG, and FFA), resulting in weight loss and reduced lipid accumulation in HFD mice. Meanwhile, DIP treatment significantly reduced the expression of lipid metabolism-related genes, including peroxisome proliferator-activated receptor γ (PPAR-γ), CCAAT/enhancer-binding protein α (C/EBPα), and sterol response element binding protein-1c (SREBP-1c). Mao et al. [72] studied the hypolipidemic activity of *Pleurotus geesteranus* 5(#) exopolysaccharide (EPS). The results showed that TC and TG concentrations in diabetic mice were reduced by 18.8% and 12.0%, respectively, after EPS treatment, and the reduction in LDL-C, HDL-C, and atherogenic index was also observed. Mfopa et al. [28] investigated the antilipidemic potential of *Ganoderma applanatum* polysaccharides (GAP) in obese rats. The results revealed that GAP intervention could reduce the rate of food intake in obese rats and thus delay the rate of weight gain, and GAP intervention at different doses could significantly reduce TC, TG, and LDL-C levels. Liu et al. [142] found that *Cordyceps taii* polysaccharides (CTP) could affect lipid metabolism parameters (TC, TG, LDL-C, and HDL) in diabetic mice -C serum level) and effectively improve lipid disorders.

### 5.3. Antitumor Activity

**Figure 13:**
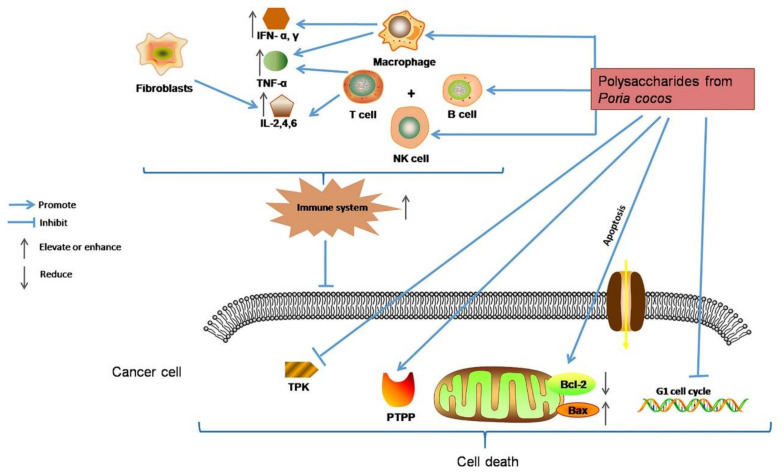
Possible antitumor mechanisms of *Poria cocos* polysaccharides [112].

As a functional food or medicine, the antitumor activity of EFPs has been thoroughly verified by numerous studies. Studies have shown that EFPs could directly attack cancer cells and also exhibit antitumor effects by activating different immune responses in the host [112]. For example, a report revealed that *Poria cocos* polysaccharides could exert their antitumor activity by overcoming the host’s stress, enhancing the killing capacity of macrophages, T cells, B cells, and NK cells by releasing cytokines to improve immunity, and directly promoting the apoptosis of tumor cells by up-regulating the expression of apoptosis-related genes (Figure 13) [112]. Wang et al. [26] selected two *Lentinus edodes* polysaccharides (SLNT1 and JLNT1) with the highest MW and the most significant antitumor activity in vitro to test their effects in hepatoma H22 cells and bearing athymic nude mice. The results showed that both SLNT1 and JLNT1 treatments could inhibit the growth of H22 tumors in mice, with the highest inhibition rates of 65.41% and 61.07%, respectively. Moreover, they could significantly increase serum IL-2 and TNF-α levels and induce tumor cell apoptosis. In another antitumor study on SLNT [158], it also effectively inhibited the proliferation of human colon cancer HT-29 cells through ROS-mediated intrinsic apoptotic pathway and TNF-α-mediated extrinsic apoptotic pathway to induce apoptosis. Consistently, SLNT treatment also could significantly inhibit the growth of colon tumors. Its regulatory mechanism may involve activating caspase-3 via intrinsic and extrinsic pathways to cause apoptosis, specifically by an intrinsic pathway of overproduction of ROS, loss of MMP, increase in Bax/Bcl-2 ratio and cytosolic cytochrome c, as well as activation of caspase-9 and -3-mediated signals, and an extrinsic pathway for increasing TNF-α level, inhibiting the NF-κB pathway, and activating caspase-8 and -3-mediated signals. Li et al. [105] used SLNT to treat tumor-bearing athymic nude mice to explore its anti-breast cancer activity. They found that SLNT treatment significantly inhibited tumor growth and increased the mouse tumor cells’ Bax/Bcl-2 protein expression ratio. SLNT could also activate the caspase-7-mediated mitochondrial apoptosis pathway in tumors and induce autophagy in tumor tissues to exert antitumor effects. In addition to autophagy, tumor cell proliferation can be inhibited by a variety of mechanisms, including cell cycle arrest, cell apoptosis, secondary necrosis, and stimulation of macrophages [159]. Niu et al. [160] isolated a novel cold water-soluble polysaccharide fraction (LGP) from *Leucopaxillus giganteus* and found that LGP could effectively protect the thymus and spleen immune organs and inhibit the proliferation of the hepatoma H22 solid tumor in a dose-dependent manner. Further studies indicated that the antitumor mechanism of LGP may be the induced apoptosis of H22 cells through S-phase arrest and the mitochondria-mediated apoptosis pathway.

### 5.4. Intestinal Homeostasis-Regulating Activity

**Figure 14:**
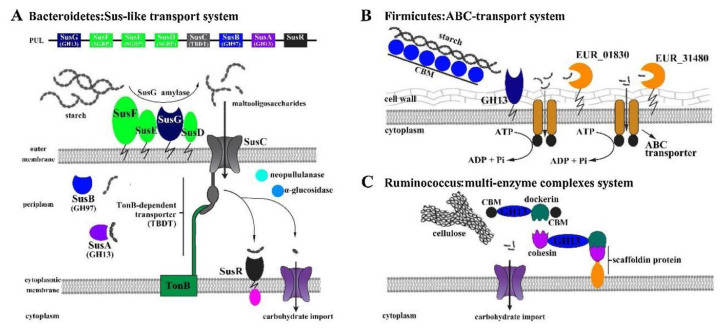
Polysaccharide degradation mechanisms of intestinal bacteria. (**A**) Starch utilization system (Sus) in *Bacteroides thetaiotaomicron* and their genomic organization of polysaccharide utilization loci (PUL). (**B**) ATP-binding cassette transport system in *Eubacterium rectale*. (**C**) Multi-enzyme complexes system in *Ruminococcus champanellensis*. Reprinted with permission from Ref. [164]. Copyright 2022, Elsevier.

**Figure 15:**
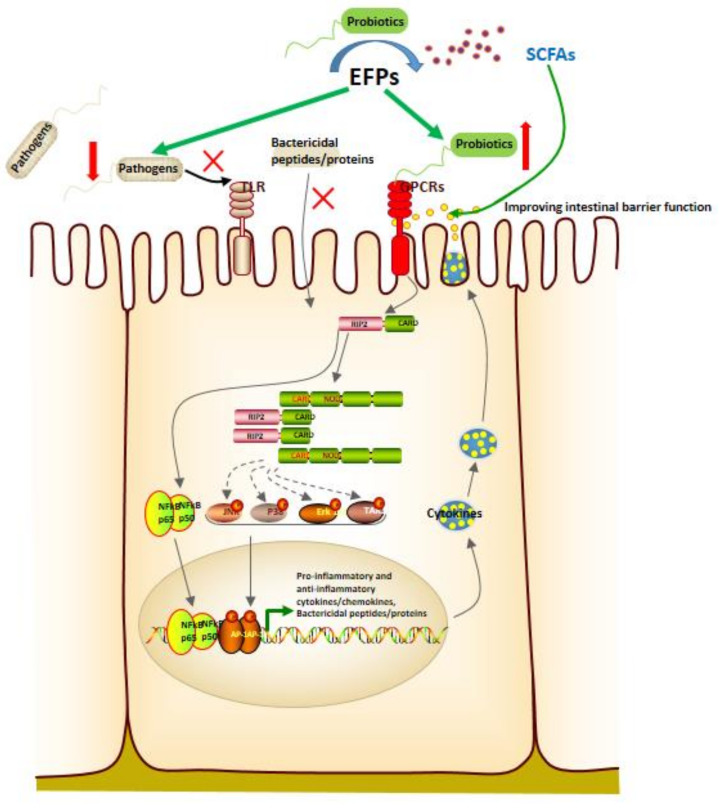
Probiotic mechanism of EFPs in intestine.

The gut microbiota is a complex and highly dynamic community of microbes that colonize the gut. It is now recognized as a promising key to determining host health [161]. The gut microbiota consists of trillions of microorganisms that perform various functions, including nutrient metabolism, maintaining intestinal pH balance, regulation of the immune system, protection from pathogen invasion, and balance of endotoxin levels [151]. The gut microbiome produces energy from food and may contribute to the development of overweight, inflammation, diabetes, and other metabolic disorders [162]. EFPs, such as chitin, α- and β-glucans, and xylan, can act as prebiotics in the gut and improve gut health by enhancing the growth of beneficial bacteria and restoring bacterial imbalances [163]. EFPs cannot be digested in the stomach or small intestine and can safely and smoothly reach the large intestine to be utilized by the intestinal flora (Figure 14) and produce beneficial metabolites such as short-chain fatty acids (SCFAs) (Figure 15), thereby improving body health [163,164]. A polysaccharide (DIP) from *Dictyophora indusiata* had been reported to reverse gut dysbiosis and increase the abundance of beneficial flora, including *Lactobacillaceae* (lactic acid-producing bacteria) and *Ruminococaceae* (butyrate-producing bacteria) [27]. By acting on the intestinal flora, it indirectly reduced LPS, TNF-α, IL-6 and IL-1β levels and increased the expression of tight junction proteins (claudin-1, occludin and zonula occludens-1) in intestinal tissue. These findings not only demonstrate a comprehensive understanding of the protective role of DIP in gut microbiota restoration but also highlight its role in enhancing gut barrier integrity, reducing inflammation, and reducing endotoxin levels. Nakahara et al. [143] investigated the effect of PEP on the gut microbiota of high-fat diet (HFD) fed mice. They found that PEPF feeding altered the gut environment of mice fed a high-fat diet, increasing the abundance of the SCFA-producing bacteria, including *Anaerostipes* and *Closterridium*, while suppressing the abundance of the *Roseburia*. In the study of Kanwal et al. [144], DIP administration restored the dysbiosis of the mouse gut microbiota induced by HFD and increased the diversity of the microbiota. At the phylum-level bacterial taxonomy, high doses of DIP resulted in a significant decrease in the abundance ratio of *Firmicutes* to *Bacteroidetes*. Khan et al. [145] used water extraction to obtain polysaccharide samples from *Ganoderma lucidum* mycelium and fruiting bodies (GL-M, GL-F, PC-M, and PC-F), and their potential prebiotic effects were evaluated. The results showed that the composition of the intestinal flora of mice was basically remodeled after treatment with polysaccharides, the diversity of OTUs was reduced, and the effect was particularly significant in the GL-F and PC-F groups. More specifically, the *Firmicutes*/*Bacteroidetes* ratio was lower in the GL-M group, and the abundance of *Actinobacteria* was significantly higher in the GL-F group. In addition, all polysaccharides significantly promoted the growth of beneficial bacteria such as *Akkermansia*, *Muciniphila*, and lactic acid-producing bacteria. Chen et al. [41] fed hyperglycemic mice with Grifola frondosa polysaccharide (GFP-N) and found that the number of *Bacteroidetes* in mouse feces was significantly increased. At the same time, the contents of *Firmicutes* and *Proteus* were significantly decreased. A decrease in *Bacteroides* and an increase in *Firmicutes* abundance are generally associated with increased dietary energy absorption and lower levels of inflammation [165], and the ratio of Bacteroides/Firmutes was positively correlated with blood glucose concentration [166]. In addition, the relative abundances of *Porphyromonas gingivalis, Akkermansia muciniphila, Lactobacillus acidophilus, Tannerella forsythia, Bacteroides acidifaciens*, and *Roseburia intestinalis* were significantly increased after GFP-N treatment. More interestingly, GFP-N treatment also triggered the interaction between the gut microbiome and host antidiabetic effects and remarkably maintained gut microbiota homeostasis. For example, the increased relative abundance of SCFA-promoting bacteria, including *Alloprevotella* and *Blautia*, can promote inflammation, insulin resistance, and T2DM remission by reducing intestinal endotoxin release into the circulation [167].

## 6. Others

In addition to the aforementioned activities, the anti-inflammatory activity of EFPs has also received increasing attention. The study by Kanwal et al. [144] showed that *Dictyophora indusiata* mushroom polysaccharide (DIP) significantly reduced the levels of pro-inflammatory cytokines such as TNF-α, IL-1β, and IL-6 in adipose and liver tissue in HFD mice in a dose-dependent manner, while enhancing anti-inflammatory cytokine expression (IL-4 and IL-10). Macrophages play a crucial role in the inflammatory response, and they release a variety of cytokines, including NO, prostaglandin mediators, and pro-inflammatory cytokines, in response to lipopolysaccharide (LPS)-induced stimuli [168]. An alkaline-extracted glucan obtained from *Inonotus obliquus* was reported to have inhibitory effects on NF-κB, COX-2, and iNOS signaling pathways in the murine macrophage RAW 264.7 cell line [146]. Smiderle et al. [147] isolated two (1→6)-β-D-glucans from *Agaricus bisporus* and *Agaricus brasiliensis* and incubated them with LPS-stimulated cells. They found that the expression of the pro-inflammatory genes, including IL-1β and COX-2, was significantly decreased. Further analysis showed that its regulation might be related to the binding of C-lectin receptors. 

Aging is a bioprocess that can cause deleterious changes in structural integrity and physiological function [169]. There are several theories about the formation of aging, including genomic mutations and the accumulation of toxic metabolites, as well as increased free radical formation [170]. Therefore, indirect anti-aging through anti-oxidation seems to be the most economically feasible. Li et al. [148] prepared acidic polysaccharides (AcAPS) and their major purified fractions (AcAPS-1, AcAPS-2, and AcAPS-3) from *Agaricus bisporus* and explored its antioxidant and anti-aging effects on aging mice. The results showed that AcAPS and its three fractions showed strong OH^−^ and DPPH free radical scavenging ability and iron ion-chelating ability in the tested concentration range, and AcAPS-2 had the more prominent capability among them. In addition, the four polysaccharides could significantly ameliorate D-galactose-induced liver damage, kidney damage and early dysregulation of lipid metabolism, and AcAPS-2 also showed stronger activity. Fang et al. [149] studied the antioxidant and anti-aging effects of *Flammulina velutipes* polysaccharide (FPS) and its sulfated-FPS (SFPS) on D-galactose-induced aging mice. Compared with FPS, SFPS had better DPPH and OH^−^ scavenging effect and iron ion-reducing power. Moreover, SFPS could also reduce lipid peroxidation (lower MDA and lipid peroxidized LPO levels) and improve the inflammatory response (lower Ach E and LPO levels) by increasing the activity of antioxidant enzymes (SOD, GSH-Px, CAT and T-AOC, NOS activities), thereby exhibiting excellent antioxidant and anti-aging effects on D-galactose-induced aging mice.

## 7. Conclusions and Prospectives

EFPs are a crucial active ingredient in mushrooms, which have received extensive attention due to their wide variety and various bioactivities. Generally, the difference in bioactivity determines the direction of product research, production, and application. In addition to the type and origin of the mushroom, another factor that affects the bioactivity of EFPs is the difference in extraction method. The authors of this paper first reviewed various extraction methods of EFPs, including the well-known and conventional HWE, UAE, and AAE. The simplicity of operation and economic viability make these three methods acceptable for extracting most EFPs. However, their extraction efficiency is relatively low, and some methods may damage the structural composition and bioactivity of EFPs, especially UAE and AAE. Therefore, improved and innovative extraction technologies such as MAE, EAE, SWE, and PEFAE have gradually emerged, which dramatically improve the extraction efficiency of EFPs and meet the demands of various functions. Out of the protection of EFPs structural integrity and their bioactivity, EAE may be a relatively optimal choice in these different extraction methods. Although the enzyme activity is easily affected and the cost is restricted, the increasingly developed enzyme engineering and its widely used application in the food, light, and pharmaceutical industries make enzyme acquisition easier. In addition, several important factors affecting enzyme activity, such as temperature, pH, dissolved oxygen, and nutritional availability, have become more controllable. Whether in the laboratory or industrial production process, these restrictions are no longer tricky. In addition, EAE can be adapted to the extraction of most EFPs because of its lower energy consumption, high efficiency, and green extraction process. Therefore, its application and development in EFP bioactivity is more popular. Different extraction methods primarily affect the polysaccharide molecular weight, glycosidic bond type, surface appearance, and helical conformation but generally do not change the monosaccharide composition. Many reports show that the immunomodulation function of EFPS is prominent, and its immunomodulatory activity seems closely related to molecular weight and monosaccharide composition. Meanwhile, numerous studies have demonstrated a strong correlation between polysaccharide antioxidant activity and high molecular weight. However, a few studies also revealed that polysaccharides with a medium or low molecular weight had superior antioxidant activity. Antioxidant activity is an important part of the active mechanism of antitumor, anti-aging, and antibacterial activities. Therefore, the relationship between the molecular weight of EFPs and their bioactivity and the relationship between monosaccharide composition and antitumor activity needs to be further explored. The chemical structure often plays a decisive role in the active function, but the overall structure of EFPs still only represents the tip of the iceberg due to the complexity of the EFP structure and the limitations of related technologies, and more innovation analysis needs to be combined in the future. Regarding bioactivity, studies have indicated that EFPs have exceptional antitumor and immunomodulatory, especially β-glucan, which is referred to as a “biological response modulator (BRM)”. Although β-glucan is not covered in this study, its excellent function in various bioactivities and its regulatory mechanism need further investigation and discussion.

## Figures and Tables

**Table 2:** Monosaccharide composition of different edible fungus polysaccharides.

Edible Fungus Origin	Monosaccharide Composition and Molar Ratio	Ref
*Cordyceps sinensis*	CP-PS, Man: Glc: Gal: xyl: Rha: Ara = 38.37: 27.44: 24.25: 5.22: 2.51:2.21, respectively	[21]
*Flammulina velutipes*	FVP2C, Glc: Gal: Man: Fuc = 100: 14: 7: 4, respectively	[22]
*Phellinus linteus*	PSCP, Glc: Man: Gal: N-Acetyl Glu: unidentified monosaccharide = 37.4: 2.2: 12.6: 29.5: 18.3, respectively	[86]
*Lentinus edodes*	SLNT1, Glc: Gly = 3.4: 1, SLNT2, Glc: Gly = 0.9: 1, JLNT1, Glc: Gly = 2.8: 1, JLNT2, Glc: Gly = 2.7: 1, JLNT2, JLNT3, Glc: Gly = 3: 1, respectively	[26]
*Dictyophora indusiate*	DP, Glc: Gal: Man = 56.2: 14.1: 29.7, respectively	[87]
*Poria cocos* Wolf	PCP-H, Man: Glc: Gal: Ara = 0.92: 0.18: 86.88: 12.01, PCP-M, Man: Glc: Gal: Ara = 4.02: 4.93: 79.48: 11.57, PCP-E, Man: Glc: Gal: Ara = 1.98: 0.36: 81.72: 15.93, PCP-U, Man: Glc: Gal: Ara = 2.18: 2.36: 87.27: 8.18, respectively	[29]
*Grifola frondosa*	GFP, L-Ara: D-Man: D-Glc = 3.79: 1.00: 49.70, respectively	[41]
*Suillellus luridus*	Suilu.A, Ara: xyl: Man: Glc: Gal = 2.06: 19.66: 46.07: 21.53, Suilu.C, Ara: xyl: Man: Glc: Gal = 2.71: 19.89: 41.90: 24.77, Suilu.S, Ara: xyl: Man: Glc: Gal = 4.36: 20.94: 49.09: 17.25, respectively	[88]
*Coprinus comatus*	EP, Man: Glu: Gal: Xyl: Ara = 6.45: 8.95: 9.64: 10.33: 11.34, IP, Man: Gal: Xyl = 6.46: 9.65: 10.35, respectively	[74]
*Oudemansiella radicata*	ORP-1, Man: Rib: Glc: Ga: xyl = 2.46: 2.63: 3.89: 3.22: 2.78, ORP-2, Rib: Glc: xyl = 2.63: 3.38: 2.65, ORP-3, Gl: xyl = 3.38: 2.65, respectively	[31]
*Paxillus involutus*	PIP2-1, Man: Glc: Gal: Fuc = 2.8: 62.2: 25.4: 9.6, respectively	[32]
*Lepista nuda*	LNP-1, Man: Glc: Gal: xyl: Ara: Fuc = 19.0: 33.5: 18.0: 4.6: 21.0: 3.9, LNP-2, Man: Glc: Gal: Ara: Fuc = 23.5: 11.4: 34.2: 21.4: 9.5, respectively	[89]

## Data Availability

Not applicable.
